# FAS: assessing the similarity between proteins using multi-layered feature architectures

**DOI:** 10.1093/bioinformatics/btad226

**Published:** 2023-04-21

**Authors:** Julian Dosch, Holger Bergmann, Vinh Tran, Ingo Ebersberger

**Affiliations:** Applied Bioinformatics Group, Goethe University Frankfurt, Faculty of Biosciences, Institute of Cell Biology and Neuroscience, Frankfurt, 60438, Germany; Applied Bioinformatics Group, Goethe University Frankfurt, Faculty of Biosciences, Institute of Cell Biology and Neuroscience, Frankfurt, 60438, Germany; Applied Bioinformatics Group, Goethe University Frankfurt, Faculty of Biosciences, Institute of Cell Biology and Neuroscience, Frankfurt, 60438, Germany; Applied Bioinformatics Group, Goethe University Frankfurt, Faculty of Biosciences, Institute of Cell Biology and Neuroscience, Frankfurt, 60438, Germany; Senckenberg Biodiversity and Climate Research Centre (S-BIKF), Frankfurt, 60325, Germany; LOEWE Centre for Translational Biodiversity Genomics (TBG), Frankfurt, 60325, Germany

## Abstract

**Motivation:**

Protein sequence comparison is a fundamental element in the bioinformatics toolkit. When sequences are annotated with features such as functional domains, transmembrane domains, low complexity regions or secondary structure elements, the resulting feature architectures allow better informed comparisons. However, many existing schemes for scoring architecture similarities cannot cope with features arising from multiple annotation sources. Those that do fall short in the resolution of overlapping and redundant feature annotations.

**Results:**

Here, we introduce FAS, a scoring method that integrates features from multiple annotation sources in a directed acyclic architecture graph. Redundancies are resolved as part of the architecture comparison by finding the paths through the graphs that maximize the pair-wise architecture similarity. In a large-scale evaluation on more than 10 000 human-yeast ortholog pairs, architecture similarities assessed with FAS are consistently more plausible than those obtained using e-values to resolve overlaps or leaving overlaps unresolved. Three case studies demonstrate the utility of FAS on architecture comparison tasks: benchmarking of orthology assignment software, identification of functionally diverged orthologs, and diagnosing protein architecture changes stemming from faulty gene predictions. With the help of FAS, feature architecture comparisons can now be routinely integrated into these and many other applications.

**Availability and implementation:**

FAS is available as python package: https://pypi.org/project/greedyFAS/.

## 1 Introduction

The sequencing of genomes from organisms representing the remotest corners of the tree of life is in full swing (Lewin et al. 2018; Mukherjee et al. 2021; Sayers et al. 2021). The toolbox to integrate the newly identified proteins into a comprehensive evolutionary and functional network is extensive. It ranges from sequence similarity-based search heuristics to identify significantly similar sequences (e.g. Altschul et al. 1997; Buchfink et al. 2014; Steinegger and Söding 2017; Potter et al. 2018) to the identification of orthologs whose evolutionary lineages split by a speciation events (e.g. Glover et al. 2019). As orthologs tend to overlap at least partly in their function, this provides at the same time a tentative functional annotation (Fang et al. 2010; Gabaldón and Koonin 2013). Sequence comparisons become more informative once proteins are annotated with features provided by PFAM (Mistry et al. 2021), SMART (Letunic et al. 2021), HAMAP (Pedruzzi et al. 2015), CDD (Lu et al. 2020), or InterPro (Blum et al. 2021). The resulting feature architectures (FAs) inform about protein function (Bashton and Chothia 2007; Forslund and Sonnhammer 2008; Kummerfeld and Teichmann 2009; Burge et al. 2012; Messih et al. 2012; Doğan et al. 2016), and thus FA comparisons should aid in the differentiation of orthologs that likely exert similar functions, and those that have functionally diverged.

Feature annotations are considered in workflows propagating functional annotations across large cohorts of homologs (e.g. Conesa and Götz 2008; Kanehisa et al. 2016; Cantalapiedra et al. 2021). These tools focus on the classification of functionally equivalent proteins. Therefore, they neither capture type nor extent of domain architecture change between homologs for use in downstream analyses that are interested, e.g. in the lineage-specific functional diversification of orthologs. Expert curation based on the visual comparisons of FAs, in turn, can quickly identify and evaluate lineage-specific architecture changes (e.g. Gerrard and Bornberg-Bauer 2003; Huang et al. 2012; Moore et al. 2014; Hsu et al. 2016). But this is limited to individual candidate proteins.

**Figure 1.**
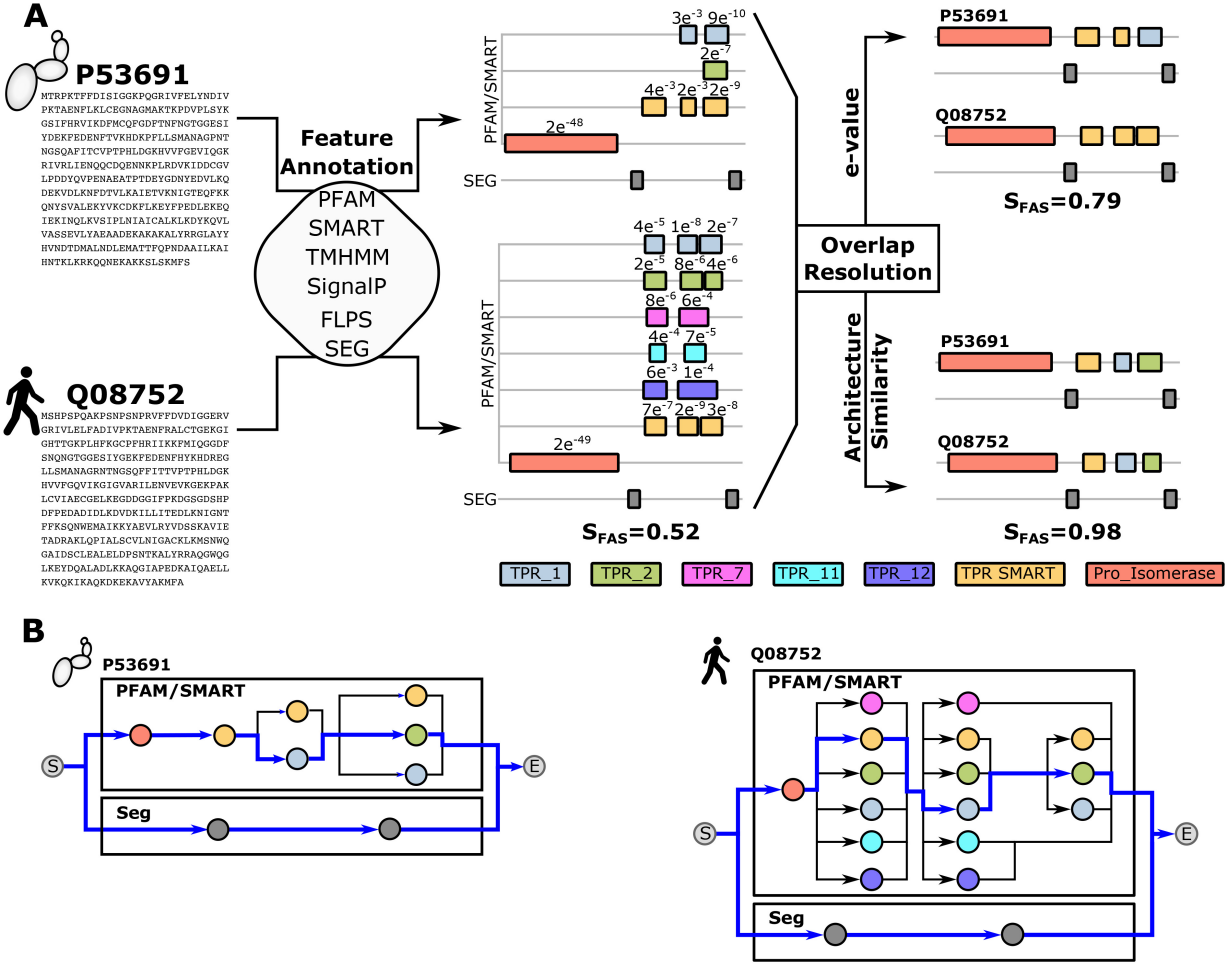
Feature annotation and architecture comparison for a functionally equivalent human-yeast ortholog pair. (A) Protein sequences of the two Peptidyl-prolyl cis-trans isomerases (PPID; EC 5.2.1.8) serve as input for feature annotation using a diverse collection of annotation tools. The resulting feature architecture reflects the assignment of each amino acid of the protein to zero, one, or in case of the tetratricopeptide repeats (TPR), many feature types. Where available, *e*-values of the feature annotations are given above the respective feature instances. The similarity score (*S*${}_{\text{FAS}}$) of the two orthologs on the feature architecture level depends on the treatment of the overlapping TPR annotations. If overlaps remain unresolved, their similarity score is only 0.52. State-of-the-art overlap resolution by selecting the feature type that was annotated with the lowest *e*-value increases the score to 0.79. The optimal overlap resolution, however, achieves a score of 0.98 (see (B)). Similarity scores were computed as given in Section 2.3. Uniprot IDs P53691—PPID${}_{\text{yeast}}$; Q08752—PPID${}_{\text{human}}$. PFAM IDs: TPR_1—PF00515; TPR_2—PF07719; TPR_7—PF13176; TPR_11—PF13414; TPR_12—PF13424; Pro_Isomerase—PF00160. SMART IDs: TPR—SM00028, (B) representation of the multi-layered feature architectures from (A) as directed acyclic graphs. Each vertex represents a feature instance and edges connect adjacent feature instances of the same annotation layer. The architectures comprise two layers, Pfam/SMART and SEG. Blue edges mark the paths through the redundant annotations in the Pfam/SMART layers that maximize the pair-wise architecture similarity

FA similarity scores can bridge the gap between large-scale automated comparisons and candidate-based visual inspections of FAs, in principle. The Jaccard index, i.e. the intersection of domains annotated in two proteins over their union, is the simplest among such measures (Geer et al. 2002). More refined scoring schemes consider additionally the extent of domain order conservation in the compared architectures. They assess the similarity in position of shared domains, the agreement in copy number for individual domains, and they optionally weigh the contribution of individual domains to the overall similarity score (Lin et al. 2006; Song et al. 2007; Lee and Lee 2009; Koestler et al. 2010; Doğan et al. 2016). Most of these approaches analyse linear FAs where each amino acid residue is assigned to one feature at most. Ambiguous assignments are typically resolved by selecting the domain with the lowest e-value (*e-value minimization*; Yeats et al. 2010; Lewis et al. 2019) (Fig. 1A). Koestler et al. (2010) devised the first scoring scheme that naturally handles overlapping domain annotation. It further allowed to include features from diverse annotation sources as additional layers into the FA. As a consequence, both sensitivity and specificity of the architecture comparison increased (Koestler et al. 2010). However, it was overlooked that this approach bears the risk of substantially underestimating architecture similarities, by that generating a spurious signal of functional diversification (see Fig. 1A). Consequently, there is still no satisfying solution for the scoring of pairwise feature architecture similarities.

Here, we introduce *FAS*, which combines the sensitivity of multi-layered FA (MLFA) comparisons with the precision of non-redundant feature annotations. Instead of resolving overlapping and redundant feature annotations *a priori*, we propose to integrate this task into the comparative analysis. This allows to resolve redundancies such that the pairwise architecture similarity is maximized. FAS achieves this by representing MLFAs as directed acyclic graphs (DAGs; Fig. 1B). Redundant annotations are represented by alternative paths through an annotation layer, and FAS resolves redundancies by identifying the highest scoring path (*score maximization*). With three example applications, we then demonstrate that FAS allows integrating feature architecture similarity assessments routinely into comparative sequence analysis.

## 2 Concept and implementation

### 2.1 Terminology

A *feature* represents a substring of protein *S*, i.e. assigned to one *feature class*, where each class comprises features from one annotation source (see Section 2.2). The union of all feature classes constitutes the *feature space*. Each feature class comprises one to many feature types. To give an example, a PFAM family represented by its profile Hidden Markov model (pHMM) is a feature type from the class ‘PFAM’. Each feature type is represented by zero to many *instances* in a protein. The union of all annotated feature instances ordered from N- to C-terminus along the protein sequence resembles the *feature architecture* of *S* where each feature class corresponds to one *layer* in the architecture (Fig. 1B). *Overlaps* in the feature architecture are generated when a sequence position is assigned to two or more types of the same feature class. If at least one overlap extends to *k* or more amino acids we consider the feature architecture as (partly) *redundant*. A representative feature architecture is the realization of a non-redundant architecture by overlap resolution that maximizes the pairwise similarity score (see Fig. 1).

### 2.2 Feature space

The default feature space of FAS comprises PFAM and SMART domains (Letunic et al. 2021; Mistry et al. 2021), transmembrane domains annotated with tmhmm (Krogh et al. 2001), low complexity regions predicted with SEG (Wootton 1994) and fLPS (Harrison 2017), and coiled coils predicted with COILS2 (Lupas 1996). Alternatively, FAS can use architectures resulting from an InterPro scan annotation (Blum et al. 2021). The feature space can be adjusted by adding/removing feature classes, and details are provided in the software manual.

### 2.3 Scoring feature architecture similarity

The FAS score is an asymmetric measure of the feature architecture similarity between a protein pair, the reference *S* and the target *O* (see Supplementary text for further information about the score asymmetry). It ranges from a minimum of 0, when the two architectures have no feature type in common, to a maximum of 1, when the reference architecture resembles the (sub-)architecture of the target. The FAS score is a linear combination of (i) the multiplicity score (MS) capturing the fraction of feature types in the architecture of the reference, i.e. represented in the target, and (ii) the positional score (PS) that captures the similarity of the position for the shared feature types in the two architectures. We compute


(1) FAS(S,O)=α⋅MS+(1−α)⋅PS

Per default, we set α = 0.7, however users can either increase α if the order of shared features is less relevant for a given analysis, or set α to lower values to increase the impact of feature order on the FAS score (see Supplementary Text and Supplementary Fig. S1). Alternatively, the MS and the PS can be inspected independently to assess how feature absence, and differences in the relative positions of shared features contribute to the dissimilarity of two architectures (Supplementary Fig. S1).

The MS and the PS scores have been introduced by Koestler et al. (2010) and will be briefly recapitulated in the following.

#### 2.3.1 Multiplicity score

We define *N* as the non-redundant set of feature types annotated in *S*, where each feature type $N_{i}$ occurs with 1 to *m* instances in the architecture of *S*. We compute the multiplicity score as where $N_{i}^{S}$ and $N_{i}^{O}$ represent the number of instances of feature type *i* in the reference and the target, respectively. If $N_{i}^{S}<N_{j}^{O}$, we apply an upper bound of 1. Each feature type is weighted by a factor $\omega_{i}$ (see Section 2.3.3) such that the MS can reach a maximum of 1.


(2) $$\text{MS}(S,O)=\sum_{i=1}^{N^{S}}\left(\omega_{i}\cdot \min\left(\frac{N_{i}^{S}\cdot N_{i}^{O}}{{\left(N_{i}^{S}\right)}^{2}},1\right)\right)$$

#### 2.3.2 Positional score

Let $P_{i,j}^{S}$ be the relative position of the *j*th instance of feature type $N_{i}$ in *S*. We then identify the corresponding instance *l* of $N_{i}$ in *O*, such that the relative distance of the two instances in *S* and *O* is minimized. We compute the positional score as $P_{i,j}^{S}$ and $P_{i,l}^{O}$ are computed as the absolute position of the feature instance midpoint in the protein sequence divided by the length of the protein. We sum the weighted partial scores for all features in *N* such that the PS reaches a maximum of 1.


(3) $$\text{PS}(S,O)=\sum_{i=1}^{N^{S}}\frac{1}{N_{i}^{S}}(\omega_{i}\sum_{j=1}^{N_{i}^{S}}(1-\underset{1\leq l\leq N_{i}^{O}}{\min}|P_{i,j}^{S}-P_{i,l}^{O}|))$$

#### 2.3.3 Domain weighting

Two weighting schemes control the influence of individual feature types on the FAS score. In the uniform weighting scheme, all feature types contribute equally to the score. This scheme can be applied if the focus lays on the architecture change itself independent of the involved feature types. In the abundance-driven weighting, the weight $\omega_{i}$ of a feature type *i* increases with its decreasing abundance in the reference proteome. This gives feature types a higher influence on the score that are less likely shared by chance (e.g. Doğan et al. 2016). Per default, we calculate where $o_{i}$ is the number of instances of feature *i* in the reference proteome, and *n* is the sum of all feature types in the currently scored path. Next to the natural logarithm we provide four additional functions (linear, log10, root4, root8) for computing $p_{i}$ where a linear transformation gives abundant features the lowest, and root8 gives the highest relative weights, respectively (Supplementary Fig. S2). However, weighting based on feature abundance cannot accommodate that individual common feature types are relevant for protein function, e.g. transmembrane domains. We therefore provide the option to ad hoc set a minimum weight for a set of features, to increase their impact on the FAS score. This allows users to customize the weighting scheme, e.g. when focussing on architecture changes involving specific feature types (Supplementary Fig. S3).


(4) $$\omega_{i}=\frac{p_{i}}{\sum_{x=1}^{n}p_{x}},\text{with }p_{i}=\frac{\sum_{l=1}^{n}\text{ln}(o_{l})+1}{\text{ln}(o_{i})+1},\text{and}\sum_{i=1}^{n}\omega_{i}=1$$

### 2.4 Resolution of redundant architectures

We represent the multi-layered feature architecture of a protein as a directed acyclic graph (Fig. 1B). Vertices denote the annotated feature instances which are connected by edges such that the order in the graph reflects the order in the protein. Per default, we connect only features of the same feature class, with one exception. Both SMART and Pfam domains are represented by pHMMs, and many SMART domains overlap with corresponding Pfam domains (Supplementary Fig. S4). Therefore, we subsume both in one feature class (option —d of FAS). A path through the graph originates then at the *start* vertex, visits vertices with increasing distance and terminates in the *end* vertex. Feature instances with an overlap larger than a pre-defined cut-off (option —max_overlap) result in alternative paths each including one of the overlapping feature types. We have implemented two search strategies to identify the path that maximizes the pair-wise FAS score.

#### 2.4.1 Exhaustive path search

During MLFA comparison, the score maximization (SM) algorithm traverses graphs in an exhaustive depth-first search. Thereby it evaluates the similarity for each pair-wise comparison of alternative paths through the reference and target architectures in the same annotation layer. The best scoring pair is then added to the representative architectures. In Fig. 1B, there are 72 alternative paths through the architecture of the human protein, and 6 paths through that of the yeast protein. Evaluating all 6×72 possible path combinations reveals that the optimal resolution of the redundant parts in the feature architecture results in a FAS score of 0.98 (see Fig. 1A).

#### 2.4.2 Priority mode

The runtime complexity of the exhaustive search scales exponentially with the number of alternative paths. This precludes the analysis of proteins with highly redundant architectures (Supplementary Fig. S5), of which Titin (Uniprot ID: Q8WZ42) with $10^{173}$ alternative paths is the most extreme example in the human protein set. We have therefore implemented an iterative search heuristic. We first resolve the redundant parts of a MLFA greedily by selecting at each graph junction the feature type that maximizes the partial FAS score up to and including the current feature instance. However, the number of instances for the selected features in the not yet visited part of the architecture graph affects both feature weighting and scoring (see Eqs. 2 and 4). Therefore, this initial step serves to obtain a lower bound for the optimal FAS score. To search for higher scoring alternative paths, we repeat the graph traversal this time assigning one feature type ‘priority’. At each junction, an instance of this feature type is given precedence, if it is present. Otherwise, FAS defaults to the greedy approach for this junction. We iterate over all feature types with multiple instances in the architecture and select the resolved architecture that maximizes the FAS score. The runtime complexity of the priority mode increases only linearly with the number of alternative paths (Supplementary Fig. S6), and FAS defaults to the priority mode when the number of possible path combinations exceeds a user-defined threshold (Default: 500).

#### 2.4.3 Input/output

FAS takes two (multi-) fasta files as input and, for each protein pair, will output their MS and PS scores, the resulting MLFA similarity score, and the overlap-resolved architectures in tsv format. Optionally, FAS can generate an output that can be directly used for visualization with PhyloProfile (Tran et al. 2018).

## 3 Materials and methods

### 3.1 Data

We downloaded pair-wise orthology assignments between human and yeast proteins (*Saccharomyces cerevisiae*) created by OMA, InParanoid, and Ensembl Compara from the OrthoBench websites (https://orthology.benchmarkservice.org/). For the frog *Xenopus tropicalis*, we downloaded five different versions of its proteome. Four versions were provided as QFO reference proteomes (releases 2019, 2020, 2020_2, 2021) (Dessimoz et al. 2012), and the fifth proteome was the annotated protein set for the NCBI RefSeq assembly GCF_000004195.4. The datasets with the corresponding sources are summarized in Supplementary Table S1.

### 3.2 Semantic similarity of GO annotation

GO term annotations (sub-ontology ‘Molecular Function’) (Carbon et al. 2021) were obtained from the Gene Ontology web sites (release 22 June 2022). Pair-wise semantic similarities of the GO annotations were computed with the simRel method (Schlicker et al. 2006) as implemented in the python package FastSemSim (https://pypi.org/project/fastsemsim/).

### 3.3 Tetrapod core gene analysis

Orthologous groups with at least nine of the following ten species represented were downloaded from the OMA database: *Homo sapiens*, *Mus musculus*, *Rattus norvegicus*, *Monodelphis domestica*, *Gallus gallus*, *Pelodiscus sinensis*, *Anolis carolinensis*, *Xenopus laevis*, *Latimeria chalumnae*, and *Ciona intestinalis*. Each of the 3460 orthologous group was then extended with orthologs from the five *X.tropicalis* proteomes using fDOG (https://www.github.com/bionf/fdog) with *X.laevis* as the reference. Average bi-directional FAS scores between all orthologs and the *X.laevis* reference protein were computed with FAS and the resulting presence-absence patterns of orthologs were visualized with PhyloProfile (Tran et al. 2018). The full profile is provided as supplementary data. To identify *X.tropicalis* proteins that differ more than expected in their MLFA from the reference, we empirically determined a FAS score cut-off for each protein. In brief, we computed for all members in the corresponding OMA ortholog group their average bi-directional FAS score with the *X.laevis* protein as the reference and computed mean and standard deviation (SD) from the resulting values. We then flagged an *X.tropicalis* protein as significantly different if its average bi-directional FAS score was more than two SD smaller than the mean.

### 3.4 Hardware and run time

Computations were run on an Intel(R) Core(TM) i5-3470 CPU @ 3.20 GHz using a single core. FAS completed overlap resolution and similarity scoring for the non-redundant list of 14 434 ortholog pairs in 23 min and 22 s resulting in an average run-time per protein pair of less than 0.1 s.

## 4 Results

FAS determines the pairwise similarity between two multi-layered feature architectures (MLFAs) resolving overlaps with a score maximization (SM) algorithm. To evaluate FAS, we used human-yeast ortholog assignments by three different ortholog search tools: OMA (2595 pairs; Altenhoff et al. 2019), InParanoid (4578 pairs; Sonnhammer and Östlund 2015), and Ensemble Compara (12 676 pairs; Yates et al. 2016). FAS scores were calculated for all orthologous pairs using default parameter values and a reference-based feature weighting.

### 4.1 Impact of overlap resolution on the architecture similarity score

**Figure 2.**
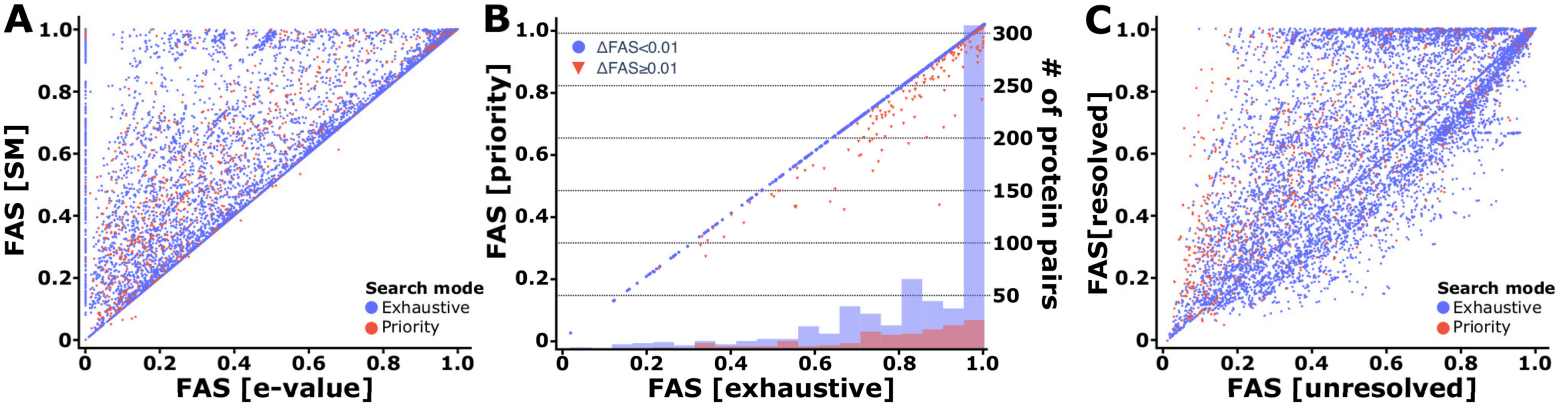
Impact of the overlap resolution on the architecture similarity assessment (A) FAS scores of human-yeast ortholog pairs resolving overlaps either with the score maximization (SM) algorithm or using a minimal *e*-value criterion. (B) For each ortholog pair, the FAS score resulting from the exhaustive path search was compared to that obtained with the priority mode. We compute ΔFAS as the difference between FAS [exhaustive] and FAS [priority]. The histogram represents the number of ortholog pairs with a ΔFAS below (blue) and above (red) 0.01 for 20 even spaced FAS score bins. (C) Comparison of FAS scores using either the resolved or the unresolved multi-layered feature architectures. For all three analyses, the mean bi-directional FAS score was used

We first investigated the effect of overlap resolution on the similarity scores using the Ensemble Compara ortholog pairs with overlaps in at least one architecture as test data (10 155 pairs; Fig. 2A). For 7032 pairs, the SM algorithm revealed in parts substantially higher similarity scores than the e-value based overlap resolution (Δ*S*${}_{\text{FAS}}$: mean = 0.20). Only in ten instances the score maximization approach resulted in a lower score. Common to all ten cases was the use of the priority mode during path search (Supplementary Table S2). Enforcing the exhaustive path search always obtained equal or higher scores than the e-value based overlap resolution.

We next assessed how often, and to what extent, the heuristic path search in priority mode resulted in an underestimation of the feature architecture similarity. We computed FAS scores for 780 human-yeast protein pairs using both the exhaustive path search and the priority mode (Fig. 2B). We selected these pairs using two criteria: the proteins had at least two alternative paths through their architectures, and the exhaustive path search could be completed within 1 h on a single CPU (approximately 10^10^ path comparisons). Most often, the results from the two search modes agree ($\Delta S_{\text{FAS}}<0.01$ for 661 pairs). In 92 cases, the priority mode resulted only in a slight underestimation ($0.01<\Delta S_{\text{FAS}}<0.1$; mean = 0.05). Only for 27 protein pairs, the FAS score computed with the priority mode was off by more than 0.1. Thus, we conclude that the priority mode gives a reasonably accurate approximation of the optimal FAS score.

In the last step, we quantified the effect of overlap resolution via the SM algorithm compared to using unresolved architectures (Fig. 2C). In most cases, overlap resolution resulted in higher FAS scores (5235; $\Delta S_{\text{FAS}}>0.01$). But surprisingly, the opposite was true for 3123 ortholog pairs. The priority mode was used only in 104 of these cases, and thus cannot explain this observation. Instead, agreeing but redundant feature types in the unresolved part of the architecture buffer the impact of a missing feature on the score (Supplementary Fig. S7). In these cases, the use of unresolved architectures results in an overestimate of architecture similarities.

Integrating all evidences reveals that FAS provides more plausible MLFA similarity scores than existing scoring schemes. We next demonstrate with three examples how MLFA comparisons can be integrated into large-scale sequence comparisons.

### 4.2 FAS scoring detects differences between ortholog search tools

**Figure 3.**
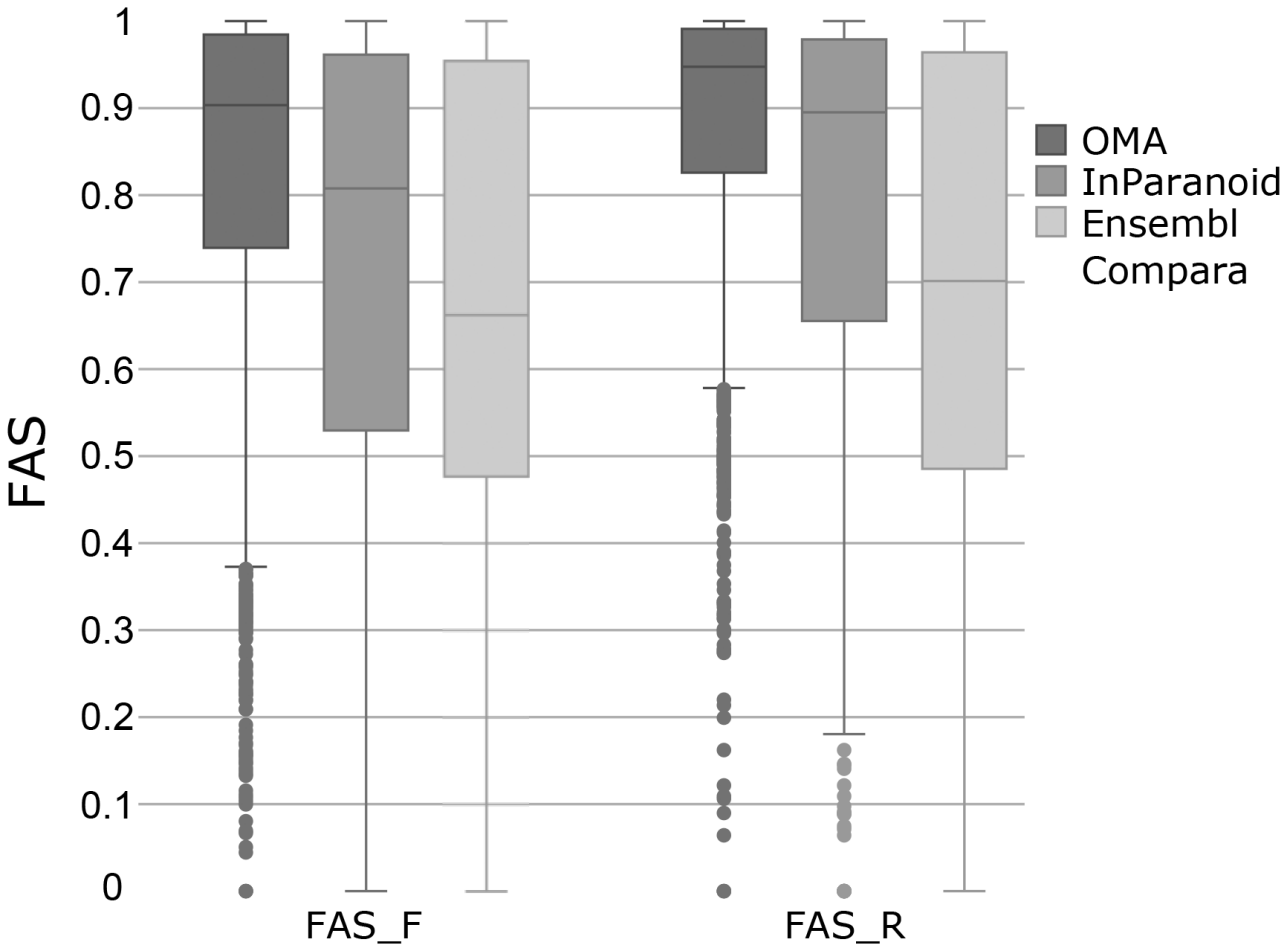
Distribution of feature architecture similarities between orthologs depends on the ortholog assignment software. FAS score distributions for human-yeast ortholog pairs assigned by OMA (2595 pairs), InParanoid (4578 pairs), and Ensembl Compara (12 676 pairs). FAS_F—human as reference; FAS_R—yeast as reference

First, we asked if and to what extent the choice of the ortholog search tool affects the architecture similarity distribution across the ortholog pairs. Because the FAS score is not symmetric (see Eqs. 2 and 3, and Supplementary text), we computed the FAS scores in both directions. We refer to FAS_F when we used the human protein as a reference and to FAS_R when we used the yeast protein as the reference (Fig. 3 and Supplementary Fig. S8A and B). Ortholog pairs identified by OMA have the highest median FAS scores (FAS_F: 0.90; FAS_R: 0.96) followed by InParanoid pairs (FAS_F: 0.80; FAS_R: 0.90). Note that the MS score penalizes the absence of reference features in the target. The higher median FAS score for the search using the yeast protein as reference therefore reveals that yeast MLFAs tend to be simpler and are often nested within more complex human architectures (see Supplementary Fig. S9). Ensembl Compara orthologs have the lowest median MLFA similarities (FAS_F = 0.62; FAS_R = 0.62). The mutually low FAS scores further indicate that both the human and the yeast proteins often harbour features in their architectures that are not represented in the ortholog of the respective other species (Supplementary Figs S10 and S11). Thus, many Ensembl Compara orthologs differ in their feature architecture to an extent, i.e. rarely seen for orthologs identified by the other two methods. However, we also note that among the 1934 human proteins for which exclusively Ensembl Compara identified a yeast ortholog, a subset has identical feature architectures in human and yeast but the sequences differ substantially in their length (Supplementary Fig. S12). In these cases, it is conceivable that minimal sequence overlap cut-offs implemented into the other two algorithms prevented the orthology assignment.

### 4.3 Low FAS scores as an indicator of functional diversification

**Figure 4.**
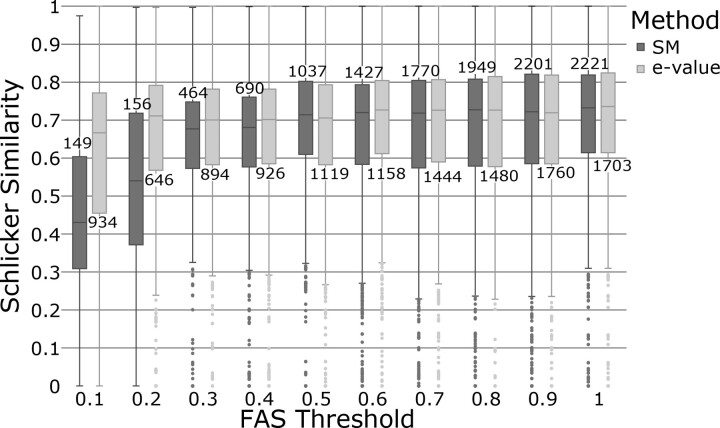
Correlation between the semantic similarity of GO annotation and the FAS score. Ensemble Compara orthologs between human and yeast were distinguished into 10 bins based on their mean bi-directional FAS score using either the score-maximization (SM) algorithm or a minimal *e*-value criterion for resolving overlapping feature types. For each bin, we plotted the semantic similarities of the GO annotations (Schlicker Score) for the pairwise orthologs as a box plot. Bin sizes are indicated above (SM) or below (*e*-value) each box plot

We next investigated to what extent feature architectures changes coincide with differences in the functional description of the orthologs. This revealed that the semantic similarities of the functional annotations (Schlicker score) decrease with decreasing FAS scores (Fig. 4), and the same trend was seen when restricted the analysis to GO terms with either evidence code EXP, or with evidence codes IBA/IBD (Supplementary Fig. S13). When we resolved overlaps in the MLFA using the minimal e-value criterion, the correlation between FAS scores and Schlicker scores vanished. However, Schlicker scores vary substantially among orthologs with the same FAS score. To track down the underlying reasons, we investigated the 80 cases where the difference between FAS and Schlicker score is 0.75 or larger in greater detail. In most cases, inaccuracies or incomplete annotations in the GO term assignment explain the score deviations (see Supplementary Text and Supplementary Table S3). Below, we present two interesting examples.

#### 4.3.1 Scenario 1: high FAS—low schlicker

Two proteins Q9Y696 (human) and Q12390 (yeast) contrast an identical feature architecture ($S_{\text{FAS}}=0.98$) with a low Schlicker score (0.01). Both proteins share the presence of two Pfam domains that are characteristic of glutathione-S-transferases (Supplementary Fig. S14). While the yeast protein is indeed annotated as a glutathione-S-transferase (GST EC 2.5.1.18; Ma et al. 2009), the human protein is a member of the chloride intracellular channel protein family (CLIC4; Supplementary Fig. S15; Littler et al. 2005). The remarkable similarities of GSTs and CLICs on the levels of domain architecture and 3D structure is known, which excludes an annotation artefact (Ponsioen et al. 2009). Instead, GSTs and CLICs are showcase examples that proteins sometimes can radically differ in function without modifying their feature architecture. However, such events seem to be rather an exception than a rule (Supplementary Text and Supplementary Table S3).

#### 4.3.2 Scenario 2: low FAS—high Schlicker

The two proteins Q7Z2Y5 (human) and Q12469 (yeast) contrast dissimilar feature architectures ($S_{\text{FAS}}=0.2$) with a high Schlicker score (0.97). Both proteins are protein kinases, they catalyse the same reaction (EC 2.7.11.1) and both are involved into cell signalling (Lin et al. 2009). What is then the relevance of the low similarity on the feature architecture level (see Supplementary Fig. S10)? The human protein, which is about 1000 amino acids longer, harbours additionally a CNH domain (SMART: SM00036), which probably acts as a regulatory domain and might be involved in macromolecular interactions (Chen et al. 1999). The yeast protein harbours additionally a P21-Rho-binding domain (PF00786) that binds Rho-like small GTPases. Its N-terminus is occupied by a Pleckstrin homology domain (PH SMART: SM00233), which is commonly found in eukaryotic signalling proteins and may play a role in recruiting proteins to different membranes (Wang and Shaw 1995). Together, these differences indicate that although both proteins catalyse the same reaction in the context of signal transduction, they very likely differ in their precise functions. This functional difference is not yet reflected in their GO annotations.

### 4.4 Feature architecture dissimilarities reveal deviating gene structure annotations

**Figure 5.**
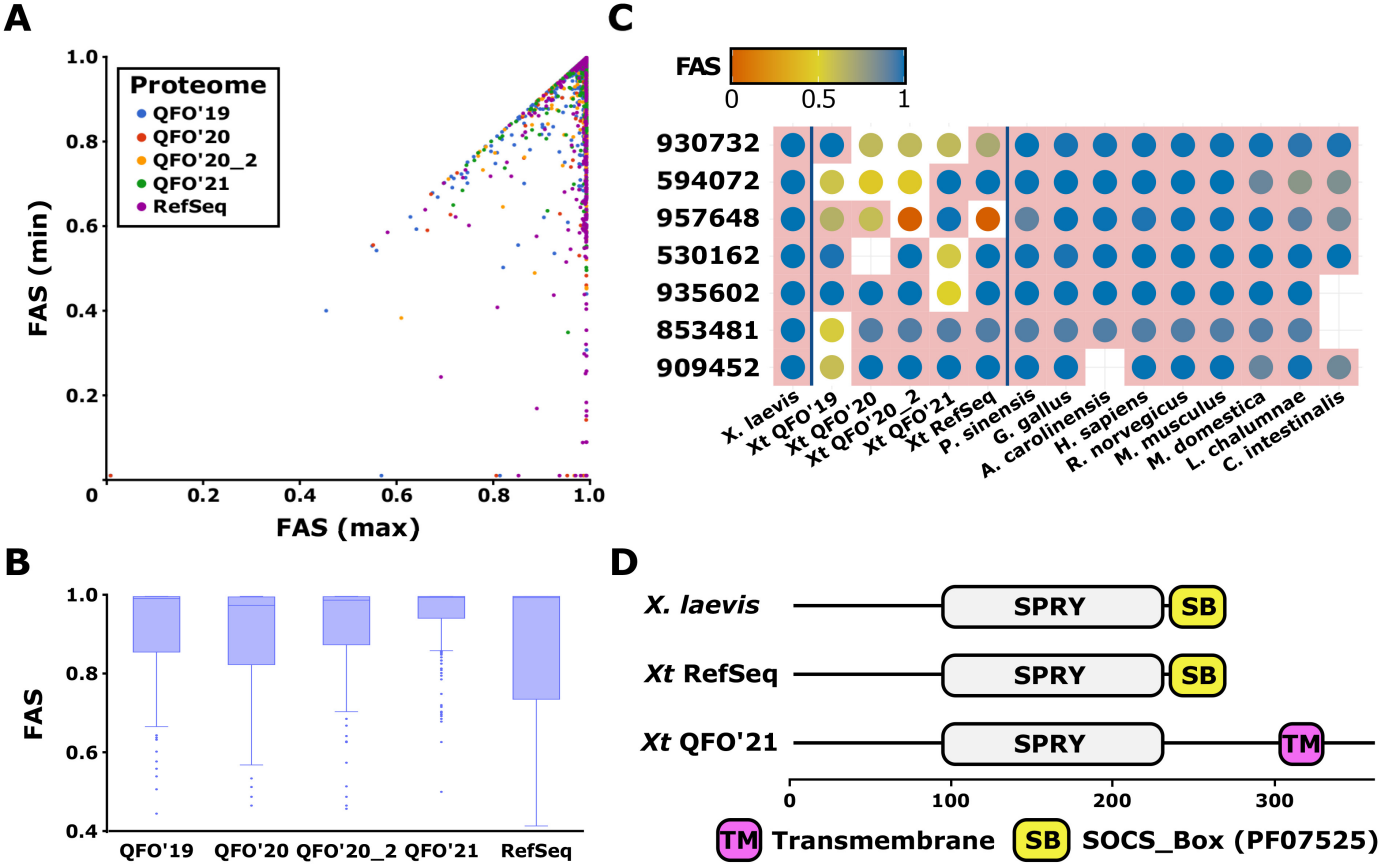
Feature architecture differences between different versions of the *X.tropicalis* proteome. (A) Scatter plot of the maximum and the minimum FAS score between the reference protein of *X.laevis* and its orthologs in the five versions of the *X.tropicalis* (*Xt*) proteome. Dot colour indicates the proteome version that provides the ortholog with the lowest FAS score. (B) FAS score distributions for the 195 *X.laevis—Xt* ortholog pairs with a FAS score difference between the highest and the lowest scoring *Xt* ortholog version of at least 0.1. (C) Phylogenetic profiles of six core genes with marked feature architecture changes between the *Xt* proteomes. Dots indicate the presence of an ortholog in the respective proteome. Dot colour informs about the mean bi-directional FAS score between the ortholog and the *X.laevis* reference protein. Dots on a white background represent orthologs that differ significantly in their feature architecture from the reference (see Section 3). Row ids represent the OMA ortholog group ids. (D) Feature architecture differences in the core group 935602. Uniprot Ids: *X.laevis*—A0A1L8FM06; Xt QFO’21—A0A6I8T262; Xt RefSeq—XP_002936603.1. The data to reproduce this analysis is provided in the Supplementary data file

The analysis of MLFAs in an evolutionary context hinges on the assumption that FA differences are not gene annotation artefacts. In the last analysis, we changed scope and applied FAS scoring to detect and evaluate MLFA differences for the same protein across different proteome versions for the same species. For the subset of 1100 tetrapod core genes with orthologs in five proteome versions of the African clawed frog *X.tropicalis*, we addressed the hypothesis that the extent of MLFA similarity between the *X.tropicalis* orthologs and the *X.laevis* reference protein is independent of the proteome version. On the first sight, the FAS score distributions show no prominent difference between the individual proteomes (Supplementary Fig. S16A; see Supplementary Fig. S16B for the data without the QFO’20 proteome). However, comparing for each core gene the minimal and the maximal FAS score obtained with the five *X.tropicalis* ortholog versions revealed, in part, substantial differences (Fig. 5A). The FAS score distributions of proteins with a difference between the highest and the lowest scoring ortholog version of at least 0.1 show that proteins from the QFO’20 reference proteome differ the most from their *X.laevis* orthologs. Thus, QFO’20 not only comprises thousands of genes less than the other proteome versions (see Supplementary Table S1) but the represented proteins are also more likely to differ in their MLFA. Both issues were largely solved with the updated release QFO’20_2. FAS scores for the QFO’21 orthologs are overall the highest whereas those from the NCBI RefSeq orthologs are most spread out (Fig. 5B and Supplementary Fig. S17). This suggests that QFO’21 is the preferred proteome for an evolutionary analysis. However, there are individual examples where the MLFA of the QFO’21 ortholog but not that of orthologs from other proteome versions deviates significantly from the *X.laevis* MLFA (Fig. 5C and D). Comparative sequences analyses investigating evolutionary change will pick up any of such gene annotation errors as candidates.

## 5 Discussion

The gold standard in interpreting architecture similarities between orthologs, or the changes thereof, is still cost- and labor-intense human curation. The scoring framework developed here aims at reducing the gap between an automatically generated pairwise FA similarity assessment and their visual interpretation of FA changes.

The main innovation in FAS is the score maximization algorithm to resolve overlapping feature types. The generation of a non-redundant feature set prior to feature annotation is one obvious alternative to the *post hoc* resolution of redundant feature annotations. CDD protein domain superfamilies result from a manually curated clustering of protein domain models that annotate overlapping footprints on protein sequences (Lu et al. 2020). Version 3.19 of this database integrates domain information from several source databases, such as Pfam v.32, SMART v6.0, COGs v1.0, TIGRFAMs v15 and Entrez Protein Clusters to form 4617 multi-model superfamilies. The integration of CDD superfamilies as an additional feature class into FAS is straightforward. However, here we have demonstrated the integration of individual source databases on the fly. FAS can thereby always use the latest release of the individual source databases, and can complement this information with custom models trained by the individual users. Moreover, it performs overlap resolution only in the context of the sequences under study.

Our study has shown that FAS is a versatile framework for the assessment of feature architecture similarity, which outperforms existing scoring schemes. This represents a major step in the direction of routinely including of FA similarity assessments into comparative analyses of protein collections whose size thus far allowed only indirect measures of architecture changes (e.g. Defosset et al. 2021). In the first example application of FAS, we have shown that the distribution of MLFA similarities between pairs of human-yeast orthologs depends on the tools that have been used for the orthology assignments. Orthology inference is an evolutionary reconstruction problem, and thus a ground truth does not exist. To still assess performance differences between individual orthology assignment tools, the Quest for Orthologs benchmark service provides several ‘challenges’ that are used to benchmark the assignments (Altenhoff et al. 2020). Two challenges are based on the assumption that orthologs tend to share a similar function (Tatusov et al. 1997). They assess, where available, the agreement in Enzyme Commission (EC) numbers and the semantic similarity in GO term annotation between ortholog pairs. However, only a fraction of proteins are enzymes, and using the semantic similarity of GO term annotations as a proxy for functional equivalence bears many pitfalls (Thomas et al. 2012; our own results). Moreover, orthologs can functionally diverge (see Supplementary Fig. S15). Similarities of feature architectures reflected in the FAS score distribution may therefore constitute an important complementary challenge in the ortholog benchmark.

The tracing of functionally equivalent orthologs across taxa is essential for a reliable protein annotation transfer (e.g. Kanehisa et al. 2016; Aramaki et al. 2020; Cantalapiedra et al. 2021). In turn, the identification of functionally diverged orthologs can help unravelling evolutionary changes that account for lineage-specific phenotypic characteristics. We could show that low FAS scores readily identify ortholog pairs with a strong indication for a functional diversification. However, errors during gene annotation such as the missing of individual exons, or the artificial fusion or fission of genes generate the same signal. While it is common practice to use protein sets from related organisms to guide the identification of genes in a newly sequenced organism (e.g. Brůna et al. 2021), testing the resulting gene predictions for consistency across taxa is not. Existing approaches focus mainly on the length of the resulting proteins but not on their feature architecture (Seppey et al. 2019). Comparing orthologs from five versions of the *X.tropicalis* proteome and up to 10 further representatives spanning the vertebrate diversity revealed proteome-specific deviations in otherwise evolutionarily highly conserved feature architectures. These instances most likely represent either artefacts of the gene annotation, or they indicate the use of an alternative isoform to represent the corresponding gene. In either case, they provide a spurious signal of lineage-specific functional diversification in a comparative sequence analysis. With the help of FAS, it is straightforward to identify and subsequently correct such errors in the annotation of protein coding genes, or alternatively to identify the isoform, i.e. most similar to the one used in other species.

### 5.1 Limits

We currently see one main challenge in the scoring of feature architecture similarities: the information gained from different feature architectures varies depending on the architecture complexity. The impact of a gain or a loss of the same feature type on the FAS score decreases with increasing number of feature types in the architecture. A case-by-case customization of feature weights can ameliorate this effect (see Supplementary Fig. S3), which might be not always feasible in large-scale analyses. Alternatively, training data can be used to infer protein-specific FAS score cut-offs below which two MLFAs can be considered significantly different. Ultimately, however, it will require manual curation to decide whether two proteins are likely to have diverged in function.

## Supplementary Material

btad226_Supplementary_Data

## Data Availability

The data underlying this article are available in the Zenondo Digital Repository, at https://doi.org/10.5281/zenodo.7896005.

## References

[btad226-B1] Altenhoff AM , Levy J, Zarowiecki M et al OMA standalone: orthology inference among public and custom genomes and transcriptomes. Genome Res 2019;29:1152–63. 31235654 10.1101/gr.243212.118 PMC6633268

[btad226-B2] Altenhoff AM , Garrayo-Ventas J, Cosentino S et al The quest for orthologs benchmark service and consensus calls in 2020. Nucleic Acids Res 2020;48:W538–W545. 32374845 10.1093/nar/gkaa308 PMC7319555

[btad226-B3] Altschul SF , Madden TL, Schäffer AA et al Gapped BLAST AND PSI-BLAST: A new generation of protein database search programs. Nucleic Acids Res 1997;25:3389–402. 9254694 10.1093/nar/25.17.3389 PMC146917

[btad226-B4] Aramaki T , Blanc-Mathieu R, Endo H et al KofamKOALA: KEGG ortholog assignment based on profile hmm and adaptive score threshold. Bioinformatics 2020;36:2251–2. 31742321 10.1093/bioinformatics/btz859 PMC7141845

[btad226-B5] Bashton M , Chothia C. The generation of new protein functions by the combination of domains. Structure 2007;15:85–99. 17223535 10.1016/j.str.2006.11.009

[btad226-B6] Blum M , Chang H-Y, Chuguransky S et al The interpro protein families and domains database: 20 years on. Nucleic Acids Res 2021;49:D344–D354. 33156333 10.1093/nar/gkaa977 PMC7778928

[btad226-B7] Brůna T , Hoff KJ, Lomsadze A et al Braker2: automatic eukaryotic genome annotation with genemark-ep+ and augustus supported by a protein database. NAR Genomics Bioinf 2021;3:lqaa108. 10.1093/nargab/lqaa108 PMC7787252 33575650

[btad226-B8] Buchfink B , Xie C, Huson DH. Fast and sensitive protein alignment using DIAMOND. Nat Methods 2015;12:59–60. 25402007 10.1038/nmeth.3176

[btad226-B9] Burge S , Kelly E, Lonsdale D et al Manual go annotation of predictive protein signatures: the interpro approach to go curation. Database J Biol Databases Curation 2012;2012:bar068. 10.1093/database/bar068 PMC3270475 22301074

[btad226-B10] Cantalapiedra CP , Hernández-Plaza A, Letunic I et al Eggnog-mapper v2: functional annotation, orthology assignments, and domain prediction at the metagenomic scale. Mol Biol Evol 2021;38:5825–9. 34597405 10.1093/molbev/msab293 PMC8662613

[btad226-B11] Carbon S , Douglass E, Good BM et al The gene ontology resource: enriching a gold mine. Nucleic Acids Research 2021;49:D325–34. 33290552 10.1093/nar/gkaa1113 PMC7779012

[btad226-B12] Chen XQ , Tan I, Leung T et al The myotonic dystrophy kinase-related Cdc42-binding kinase is involved in the regulation of neurite outgrowth in PC12 cells. J Biol Chem 1999;274:19901–5. 10391936 10.1074/jbc.274.28.19901

[btad226-B13] Conesa A , Götz S. Blast2GO: a comprehensive suite for functional analysis in plant genomics. Int J Plant Genomics 2008;2008:1. 10.1155/2008/619832 PMC2375974 18483572

[btad226-B14] Defosset A , Kress A, Nevers Y et al Proteome-scale detection of differential conservation patterns at protein and subprotein levels with BLUR. Genome Biol Evol 2021;13:evaa248. 33211099 10.1093/gbe/evaa248 PMC7851591

[btad226-B15] Dessimoz C , Gabaldón T, Roos DS, Quest for Orthologs Consortium et al Toward community standards in the quest for orthologs. Bioinformatics 2012;28:900–4. 22332236 10.1093/bioinformatics/bts050 PMC3307119

[btad226-B16] Doğan T , MacDougall A, Saidi R et al UniProt-DAAC: domain architecture alignment and classification, a new method for automatic functional annotation in UniProtKB. Bioinformatics 2016;32:2264–71. 27153729 10.1093/bioinformatics/btw114 PMC4965628

[btad226-B17] Fang G , Bhardwaj N, Robilotto R et al Getting started in gene orthology and functional analysis. PLoS Comput Biol 2010;6:e1000703. 20361041 10.1371/journal.pcbi.1000703 PMC2845645

[btad226-B18] Forslund K , Sonnhammer ELL. Predicting protein function from domain content. Bioinformatics 2008;24:1681–7. 18591194 10.1093/bioinformatics/btn312

[btad226-B19] Gabaldón T , Koonin EV. Functional and evolutionary implications of gene orthology. Nat Rev Genet 2013;14:360–6. 23552219 10.1038/nrg3456 PMC5877793

[btad226-B20] Geer LY , Domrachev M, Lipman DJ et al CDART: protein homology by domain architecture. Genome Res 2002;12:1619–23. 12368255 10.1101/gr.278202 PMC187533

[btad226-B21] Gerrard DT , Bornberg-Bauer E. Domosaic – analysis of the mosaic-like domain arrangements in proteins. Informatica (Ljubljana) 2003;27:15–20.

[btad226-B22] Glover N , Dessimoz C, Ebersberger I et al Advances and applications in the quest for orthologs. Mol Biol Evol 2019;36:2157–64. 31241141 10.1093/molbev/msz150 PMC6759064

[btad226-B23] Harrison PM. FLPS: fast discovery of compositional biases for the protein universe. BMC Bioinformatics 2017;18:476. 29132292 10.1186/s12859-017-1906-3 PMC5684748

[btad226-B24] Hsu C-H , Chiang AWT, Hwang M-J et al Proteins with highly evolvable domain architectures are nonessential but highly retained. Mol Biol Evol 2016;33:1219–30. 26769031 10.1093/molbev/msw006

[btad226-B25] Huang Q-S , Xie X-L, Liang G et al The gh18 family of chitinases: their domain architectures, functions and evolutions. Glycobiology 2012;22:23–34. 21750098 10.1093/glycob/cwr092

[btad226-B26] Kanehisa M , Sato Y, Morishima K et al Blastkoala and ghostkoala: kegg tools for functional characterization of genome and metagenome sequences. J Mol Biol 2016;428:726–31. 26585406 10.1016/j.jmb.2015.11.006

[btad226-B27] Koestler T , von Haeseler A, Ebersberger I et al Fact: functional annotation transfer between proteins with similar feature architectures. BMC Bioinformatics 2010;11:417. 20696036 10.1186/1471-2105-11-417 PMC2931517

[btad226-B28] Krogh A , Larsson B, von Heijne G et al Predicting transmembrane protein topology with a hidden markov model: application to complete genomes. J Mol Biol 2001;305:567–80. 11152613 10.1006/jmbi.2000.4315

[btad226-B29] Kummerfeld SK , Teichmann SA. Protein domain organisation: adding order. BMC Bioinformatics 2009;10:39. 19178743 10.1186/1471-2105-10-39 PMC2657131

[btad226-B30] Lee B , Lee D. Protein comparison at the domain architecture level. BMC Bioinformatics 2009;10:S5. 10.1186/1471-2105-10-S15-S5 PMC2788356 19958515

[btad226-B31] Letunic I , Khedkar S, Bork P et al SMART: recent updates, new developments and status in 2020. Nucleic Acids Res 2021;49:D458–D460. 33104802 10.1093/nar/gkaa937 PMC7778883

[btad226-B32] Lewin HA , Robinson GE, Kress WJ et al Earth biogenome project: Sequencing life for the future of life. Proc Natl Acad Sci U S A 2018;115:4325–33. 29686065 10.1073/pnas.1720115115 PMC5924910

[btad226-B33] Lewis TE , Sillitoe I, Lees JG et al Cath-resolve-hits: a new tool that resolves domain matches suspiciously quickly. Bioinformatics 2019;35:1766–7. 30295745 10.1093/bioinformatics/bty863 PMC6513158

[btad226-B34] Lin K , Zhu L, Zhang D-Y et al An initial strategy for comparing proteins at the domain architecture level. Bioinformatics 2006;22:2081–6. 16837531 10.1093/bioinformatics/btl366

[btad226-B35] Lin M , Unden H, Jacquier N et al The Cdc42 effectors Ste20, Cla4, and Skm1 down-regulate the expression of genes involved in sterol uptake by a mitogen-activated protein kinase-independent pathway. MBoC 2009;20:4826–37. 19793923 10.1091/mbc.E09-01-0034 PMC2777111

[btad226-B36] Littler DR , Assaad NN, Harrop SJ et al Crystal structure of the soluble form of the redox-regulated chloride ion channel protein CLIC4. FEBS J 2005;272:4996–5007. 16176272 10.1111/j.1742-4658.2005.04909.x

[btad226-B37] Lu S , Wang J, Chitsaz F et al CDD/sparcle: the conserved domain database in 2020. Nucleic Acids Res 2020;48:D265–D268. 31777944 10.1093/nar/gkz991 PMC6943070

[btad226-B38] Lupas A. [30] Prediction and analysis of coiled-coil structures. Methods Enzymol 1996;266:513–525. 8743703 10.1016/s0076-6879(96)66032-7

[btad226-B39] Ma X‐X , Jiang Y‐L, He Y‐X et al Structures of yeast glutathione-s-transferase gtt2 reveal a new catalytic type of GST family. EMBO Rep 2009;10:1320–6. 19851333 10.1038/embor.2009.216 PMC2799204

[btad226-B40] Messih MA , Chitale M, Bajic VB et al Protein domain recurrence and order can enhance prediction of protein functions. Bioinformatics 2012;28:i444–i450. 22962465 10.1093/bioinformatics/bts398 PMC3436825

[btad226-B41] Mistry J , Chuguransky S, Williams L et al Pfam: the protein families database in 2021. Nucleic Acids Res 2021;49:D412–D419. 33125078 10.1093/nar/gkaa913 PMC7779014

[btad226-B42] Moore AD , Held A, Terrapon N et al Domosaics: software for domain arrangement visualization and domain-centric analysis of proteins. Bioinformatics 2014;30:282–3. 24222210 10.1093/bioinformatics/btt640

[btad226-B43] Mukherjee S , Stamatis D, Bertsch J et al Genomes online database (GOLD) v.8: overview and updates. Nucleic Acids Res 2021;49:D723–33. 33152092 10.1093/nar/gkaa983 PMC7778979

[btad226-B44] Pedruzzi I , Rivoire C, Auchincloss AH et al Hamap in 2015: updates to the protein family classification and annotation system. Nucleic Acids Res 2015;43:D1064–D1070. 25348399 10.1093/nar/gku1002 PMC4383873

[btad226-B45] Ponsioen B , van Zeijl L, Langeslag M et al Spatiotemporal regulation of chloride intracellular channel protein CLIC4 by RHOA. MBoC 2009;20:4664–72. 19776349 10.1091/mbc.E09-06-0529 PMC2777097

[btad226-B46] Potter SC , Luciani A, Eddy SR et al HMMER web server: 2018 update. Nucleic Acids Res 2018;46:W200–W204. 29905871 10.1093/nar/gky448 PMC6030962

[btad226-B47] Sayers EW , Cavanaugh M, Clark K et al Genbank. Nucleic Acids Res 2021;49:D92–D96. 33196830 10.1093/nar/gkaa1023 PMC7778897

[btad226-B48] Schlicker A , Domingues FS, Rahnenführer J et al A new measure for functional similarity of gene products based on gene ontology. BMC Bioinformatics 2006;7:302. 16776819 10.1186/1471-2105-7-302 PMC1559652

[btad226-B49] Seppey M , Manni M, Zdobnov EM. BUSCO: Assessing genome assembly and annotation completeness. Methods Mol Biol 2019;1962:227–45. 31020564 10.1007/978-1-4939-9173-0_14

[btad226-B50] Song N , Sedgewick RD, Durand D et al Domain architecture comparison for multidomain homology identification. J Comput Biol 2007;14:496–516. 17572026 10.1089/cmb.2007.A009

[btad226-B51] Sonnhammer EL , Östlund G. InParanoid 8: orthology analysis between 273 proteomes, mostly eukaryotic. Nucleic Acids Res 2015;43:D234–D239. 25429972 10.1093/nar/gku1203 PMC4383983

[btad226-B52] Steinegger M , Söding J. MMseqs2 enables sensitive protein sequence searching for the analysis of massive data sets. Nat Biotechnol 2017;35:1026–8. 29035372 10.1038/nbt.3988

[btad226-B53] Tatusov RL , Koonin EV, Lipman DJ et al A genomic perspective on protein families. Science (New York, N.Y.) 1997;278:631–7. 9381173 10.1126/science.278.5338.631

[btad226-B54] Thomas PD , Wood V, Mungall CJ, on behalf of the Gene Ontology Consortium et al On the use of gene ontology annotations to assess functional similarity among orthologs and paralogs: a short report. PLoS Comput Biol 2012;8:e1002386. 22359495 10.1371/journal.pcbi.1002386 PMC3280971

[btad226-B55] Tran N-V , Greshake Tzovaras B, Ebersberger I et al PhyloProfile: dynamic visualization and exploration of multi-layered phylogenetic profiles. Bioinformatics 2018;34:3041–3. 29659708 10.1093/bioinformatics/bty225

[btad226-B56] Wang DS , Shaw G. The association of the C-terminal region of beta I sigma II spectrin to brain membranes is mediated by a PH domain, does not require membrane proteins, and coincides with a inositol-1,4,5 triphosphate binding site. Biochem Biophys Res Commun 1995;217:608–15. 7503742 10.1006/bbrc.1995.2818

[btad226-B57] Wootton JC. Non-globular domains in protein sequences: automated segmentation using complexity measures. Comput Chem 1994;18:269–85. 7952898 10.1016/0097-8485(94)85023-2

[btad226-B58] Yates A , Akanni W, Amode MR et al Ensembl 2016. Nucleic Acids Res 2016;44:D710–D716. 26687719 10.1093/nar/gkv1157 PMC4702834

[btad226-B59] Yeats C , Redfern OC, Orengo C et al A fast and automated solution for accurately resolving protein domain architectures. Bioinformatics 2010;26:745–51. 20118117 10.1093/bioinformatics/btq034

