# Supplementary material for: FAS: assessing the similarity between proteins using multi-layered feature architectures
Source: Bioinformatics. 2023 Apr 21;39(5):btad226. doi: 10.1093/bioinformatics/btad226 (PMC10185405; doi:10.1093/bioinformatics/btad226)
Supplement: btad226_Supplementary_Data [file btad226_supplementary_data.zip › Dosch_SupplementaryMaterial-r1_nomark.pdf]

Supplementary Material for

FAS: Assessing the similarity between proteins using multi-layered feature architectures

Julian Dosch[1], Holger Bergmann[1], Vinh Tran[1], and Ingo Ebersberger[1,2,3]

1 Goethe University Frankfurt, Faculty of Biosciences, Institute of Cell Biology and Neuroscience, Frankfurt, Germany

2 Senckenberg Biodiversity and Climate Research Centre (S-BIKF), Frankfurt, Germany

3 LOEWE Centre for Translational Biodiversity Genomics (TBG), Frankfurt, Germany

Supplementary material 3

Supplementary text 3
Asymmetry of the FAS score 3
Impact of the multiplicity score and the positional score on the final FAS score 3
Discrepancy between FAS and Schlicker scores 4
Empirical decision on a FAS score cutoff 5

Supplementary Figures 6
Figure S1: Correlation between the Multiplicity Score and the Positional Score. 6
Figure S2: Impact of the weighting function on the feature weights. 7
Figure S3: Impact of a customized weighting of transmembrane domains on the FAS score. 7
Figure S4: Overlap analysis of Pfam and SMART domains. 8
Figure S5: Distribution of MLFA graph complexities across the human protein set. 9
Figure S6: Path search complexity of the exhaustive vs. the priority mode. 9
Figure S7: Redundant feature annotations buffer the effect of missing domains. 10
Figure S8: Analysis of the asymmetry between the forward (FAS_F) and the reverse FAS score (FAS_R). 11
Figure S9: An example of nested feature architectures. 12
Figure S10: Feature architectures of Q7Z2Y5 and Q12469. 13
Figure S11: FAS score similarity distribution depends on the ortholog predictor. 14
Figure S12: Characterization of 1,934 human proteins for which only Ensembl Compara assigned a yeast ortholog. 15
Figure S13: Semantic similarity of GO annotations decreases with decreasing feature architecture similarity. 15
Figure S14: Feature architectures of Q9Y696 and Q12390. 16
Figure S15: CD search result for Q9Y696 and Q12390. 17
Figure S16: FAS score distribution between the *X. laevis* reference proteins and their orthologs in the *X. tropicalis* proteome versions 18
Figure S17: Feature architecture differences between different versions of the Xenopus tropicalis proteome without QFO'20. 19
Figure S18: Feature architectures of Q5TAP6 and Q04500 19
Figure S19: Examples for proteins that share the same GO annotation despite pronounced differences in their feature architectures. 20

Supplementary tables 21
Table S1: Proteome sources 21
Table S2: Path complexities of the 10 human-yeast ortholog pairs for which the score maximization algorithm to resolve overlaps resulted in a lower score than the e-value-based approach 21
Table S3: Curation of 80 human-yeast ortholog pairs with substantial discrepancies between FAS and Schlicker scores (|FAS – Schlicker| >= 0.75) 21
Table S4: Molecular Function GO term annotation for the two ortholog groups (A1A4F0 & Q06328, P38279, Q12010) and (Q5TAP6 & Q04500) 22

Supplementary data 22

References 22

Supplementary material

Supplementary text

Asymmetry of the FAS score

The FAS score reflects the similarity between two feature architectures, but its value depends on the direction of the comparison. Using a human – yeast protein pair as an example, we compute the FAS_F score with the human protein as the reference, and the FAS_R score with the yeast protein as the reference. If the two architectures differ due to features confined to the human architecture, the FAS_F score will be smaller than one, whereas the FAS_R score will remain close or equal to one (see C1 in Fig. S8). The reverse applies to differences due to features confined to the target architecture (see C2 in Fig. S8). As one example, C1 and C2 are typical for architecture differences due to gene fusions and gene fissions, irrespective of whether they represent evolutionary events or artifacts of the gene prediction. If, however, both architectures harbor exclusive features, both FAS_F and FAS_R will reflect the differences (see C3 in Fig. S8). Thus, the asymmetry of the FAS score provides the user not only with the information that two architectures differ, but also about the direction of the change.

For the above reasons, we recommend computing and analyzing both scores. Option --*reciprocal* computes the scoring in both directions, and the scores can be viewed at in a scatterplot (see Fig. S8), or alternatively in the context of 'feature architecture-aware phylogenetic profiles' using the PhyloProfile software.

If a single similarity score is required, we typically recommend computing the average bi-directional FAS score as (FAS_F + FAS_R)x0.5. This allows deciding whether two architectures differ, although information about the directionality of the change is lost. In the main manuscript, we demonstrate the separate consideration of FAS-F and FAS-R in Fig. 3, and of the average bi-directional score in Figs. 2, 4 and 5. However, depending on the scope of the analysis, alternatively either the minimum or the maximum FAS score may be used as well.

Impact of the multiplicity score and the positional score on the final FAS score

The FAS score is a weighted linear combination of the multiplicity score (MS) and the positional score (PS) where the weight α determines the contribution of the MS, and (1- α) determines the contribution of the PS. Per default, we set $\alpha = 0.7$ but the user can adjust this parameter. We cannot give an all-embracing guide of how to select an optimal value, but Fig. S1 shows the correlation between the two scores for the human-yeast orthologs inferred by OMA. This reveals

that the two scores are highly correlated, and thus the choice of the relative weights for the MS and the PS has no substantial effect on the scoring (see Fig. S1A). This is not surprising, because the way how OMA infers orthologs is tightly linked to co-linearity (alignability) of the sequences. However, if the use case is to detect protein pairs (either orthologous, paralogous, or without a detectable homology) that agree both in the feature content AND in the order of the features, the positional score will become increasingly important. We demonstrate this in Fig. S1B, where we took the ortholog pairs from Fig. S1A and randomly shuffled the feature order without creating novel overlaps. Figs S1C-D show that only the PS captures this change. It is now up to the user to decide on the relevance of feature order, and this can be adjusted with the values of α (see Fig S1E-G). However, please note that FAS provides also the values for the MS and the PS separately in its output, and thus the user can explore the two scores independent of any ad-hoc weighting.

Discrepancy between FAS and Schlicker scores

A high feature architecture similarity is typically considered as evidence that two evolutionarily related proteins are at least similar in their function. In turn, pronounced differences in the feature architectures should indicate a functional diversification. We evaluated to what extent the FAS score that reflects the pairwise similarity between two multi-layered feature architectures and the Schlicker score that reflects the semantic similarity of the functional annotation with GO terms that have been assigned to the two proteins are correlated. While we see a strong trend that proteins with a low FAS score tend to have a low Schlicker score (see Fig. 4 in main text) there is a surprisingly high variation within the individual FAS bins. We evaluated 80 examples with an extreme difference between the two scores. In most cases, limitations in the GO annotation can explain the difference (see Table S3 and main text), and we discuss here two examples in greater detail.

Scenario 1: High FAS – low Schlicker scores. Agreeing feature architectures are often taken as evidence that two orthologs share the same or at least a similar function. It is therefore surprising that Q5TAP6 (human; gene name: UTP14C) and Q04500 (yeast; gene name: UTP14) contrast a high FAS score (0.90) with a Schlicker score of only 0.06. The feature architecture of the yeast protein differs only in the presence of an N-terminal D rich extension from that of its human ortholog (Fig. S18). In line with their conserved architectures, both proteins are annotated as a U3 small nucleolar RNA-associated protein 14, and both are components of the small subunit

processome (Black et al., 2018) (human - https://www.genenames.org/data/gene-symbol-report/#!/hgnc_id/HGNC:20321). Thus, there is no indication that the two proteins have diverged in function. Instead, the low Schlicker score indicates that the GO annotations of the two proteins does not yet reflect their functional equivalence (Tables S3 and S4).

Scenario 2: Low FAS – high Schlicker scores. Substantial differences in the feature architectures indicate a functional diversification, whereas high Schlicker scores suggest the opposite. Three yeast proteins, Q06328, P38279 and Q12010, are annotated as amino acid transmembrane transporters. Accordingly, their MLFAs harbor several transmembrane domains (Fig. S19, Tables S3 and S4). The human ortholog A1A4F0 is less than half the size of the yeast proteins and its MLFA is devoid of any features, which results in a FAS score of 0. Despite the lack of transmembrane domains, A1A4F0 is annotated with GO terms associated with amino acid transmembrane transport (Evidence code IBA: inferred from biological aspect of ancestor; ECO:0000318). This explains the high semantic similarities of the GO annotations for these protein pairs (0.97), which must be considered as spurious according to our evidence.

Empirical decision on a FAS score cutoff

The FAS score is a sequence similarity measure, and like for other sequence similarity measures, e.g. percent sequence identity, a link to functional (dis-)similarity exists. But the exact value below which the similarity of two homologs becomes suspicious, changes case-by-case. We suggest two pragmatic approaches (next to the visual inspection of the architectures):

First, select an ad hoc cutoff value that is small enough such that a functional divergence (or an annotation artefact see Fig. 5 in the main text) becomes likely. For example, Fig. 4 in the main manuscript shows that even the very insensitive Schlicker score begins to decrease once FAS scores fall below 0.5, which would make a FAS score cutoff of 0.5 a pragmatic, yet very stringent solution.

Second, use training data to determine a protein-specific empirical FAS score cutoff. Select a set of well annotated proteins e.g., a KEGG ortholog group if a direct link to protein function is desired, or alternatively an OMA ortholog group, and perform the following steps: Determine the pair-wise FAS scores—we use the mean of FAS_F and FAS_R—from the training data, compute mean and standard deviation (SD), and flag any test sequence as potentially functionally diverged if its FAS score is more than 2 SD smaller than the mean. This approach is used in the analysis underlying Fig. 5 using members of OMA ortholog groups as training data.

Supplementary Figures

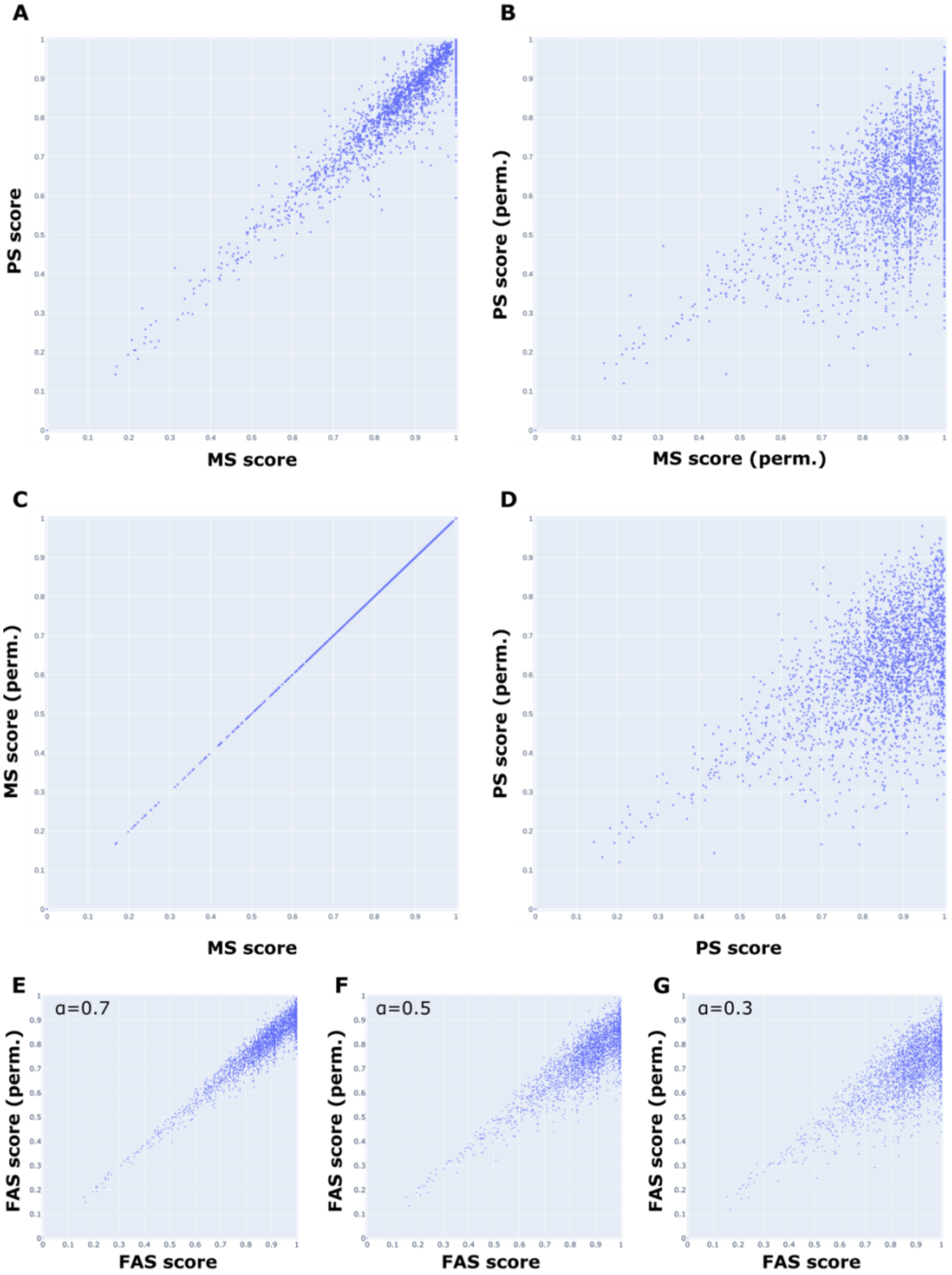

Figure S1: Correlation between the Multiplicity Score and the Positional Score. (A) For each human-yeast ortholog pair, we plotted the Multiplicity Score (MS) against the Positional Score (PS). The scatter plot shows that the two scores are highly correlated. (B) For each pair from (A), we randomized the feature order in the human feature architecture and plotted the resulting MS (perm.) and PS (perm.). While this leaves the MS score unaffected (C), the PS score drops markedly (D). The impact of the permutation on the FAS score is shown for different values of α in panels E-F.

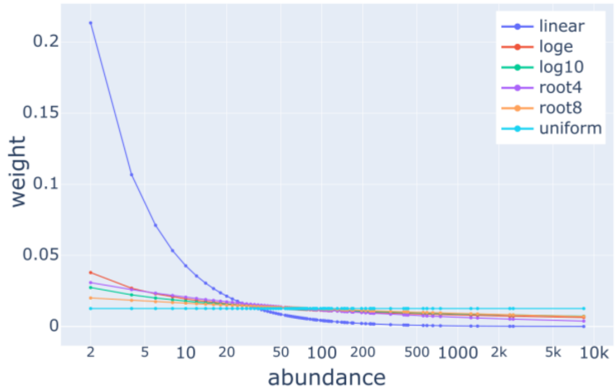

Figure S2: Impact of the weighting function on the feature weights. FAS weighs the contribution of a feature to the similarity score based on the number of proteins in the reference proteome carrying at least one instance of a feature type (feature abundance). Six different weighting functions are currently implemented (see plot inlay). Linear weighting results in a highly biased score distribution with rare features having a very high weight. Uniform weighting, in turn, renders the feature weight independent of the feature abundance, and thus gives all features the same weight.

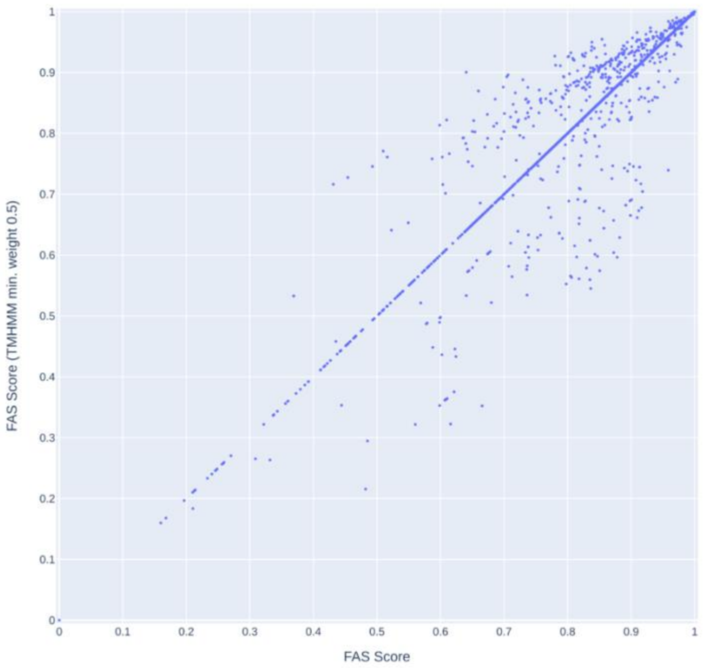

Figure S3: Impact of a customized weighting of transmembrane domains on the FAS score. Fig. S3 – FAS scores of human – yeast ortholog pairs computed with the default abundance-based weighing (x-axis) and with a scheme where the minimum weight of transmembrane domains

(tmhmm) was set to 0.5 (y-axis). Off-diagonal dots represent proteins with transmembrane domains whose orthologs differ in their feature architectures. If the difference involves the transmembrane domain the corresponding dot will be placed below the diagonal, otherwise it is placed above the diagonal.

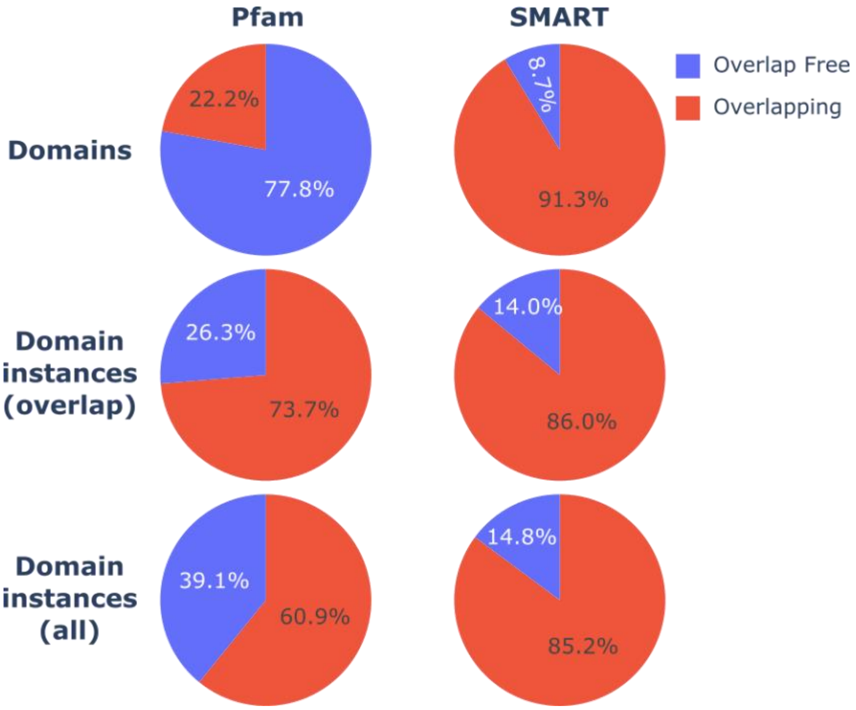

Figure S4: Overlap analysis of Pfam and SMART domains. We used the function Fas.overlapStatistics to investigate the extent to which Pfam and SMART domains overlap (at least 80% of the longer domain needs to be covered by the overlap). This reveals that only 22% of the Pfam domains overlap with a SMART domain (top left), whereas 91% of the SMART domains overlap with a Pfam domain (top right) in at least one human protein. The middle and the bottom row give the same information, however on the level of the individual domain instances. Middle row – only instances of domains with at least one overlap (red wedges from top row) are considered; bottom row – instances of all domains. The bottom row reveals that Pfam domains with an overlap to a SMART domain are represented by more feature instances in the human proteome than Pfam domains without an overlap.

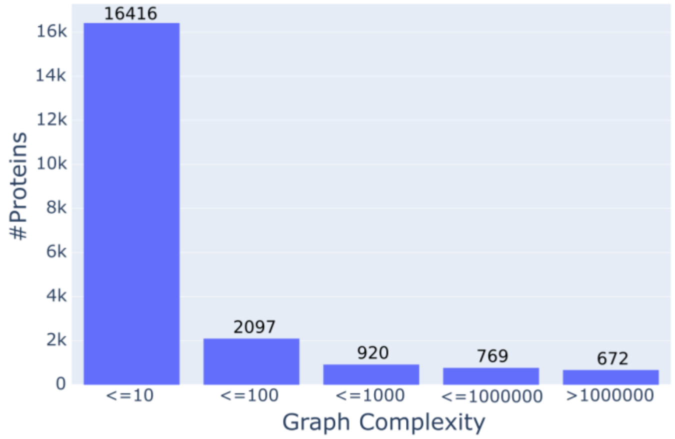

Figure S5: Distribution of MLFA graph complexities across the human protein set. The histogram gives for the human proteins in the QFO2020 reference proteome the number of alternative paths (graph complexity) through redundant parts of their MLFAs. More than 672 proteins have more than 1 million alternative paths. Of these, the human protein Titin (Uniprot ID: Q8WZ42) with 10^{173} alternative paths is the most extreme example.

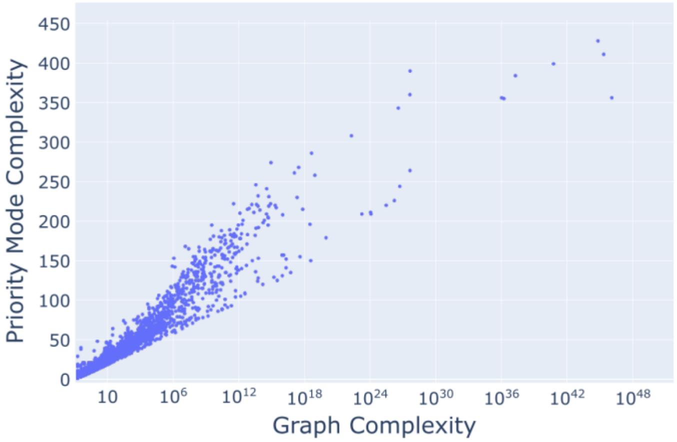

Figure S6: Path search complexity of the exhaustive vs. the priority mode. Each dot represents a human protein. The x-axis gives the number of possible representative paths through overlapping parts of the feature architecture (Pfam-/SMART layer). The y-axis gives, for the same protein, the number of paths that have to be evaluated when using the priority mode for finding the path that maximizes the FAS score.

Q6IQ22 (Human)

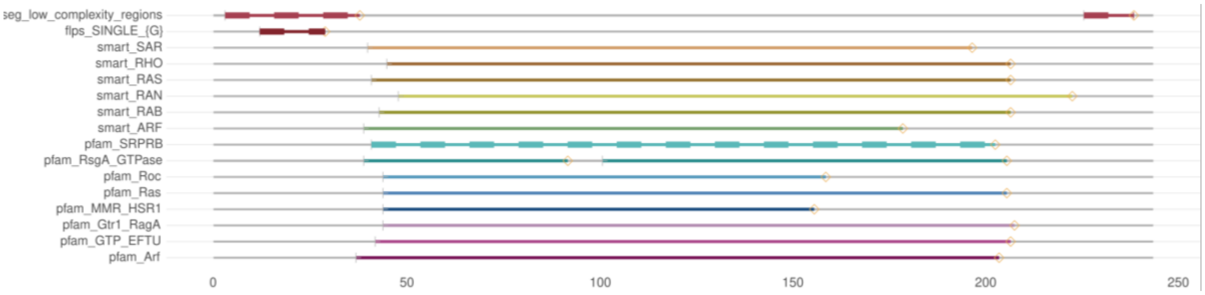

Q99260 (Yeast)

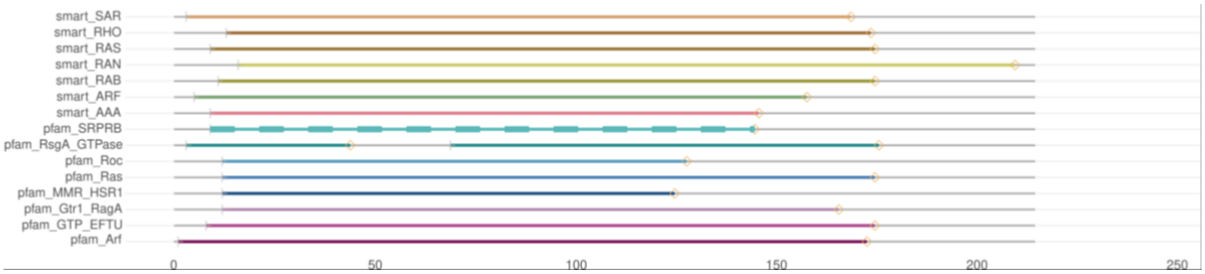

Figure S7: Redundant feature annotations buffer the effect of missing domains. The central parts of Q61Q22 (Human) and of Q99260 (Yeast) are congruently annotated with a multitude of overlapping Pfam- and SMART domains. When these redundant feature annotations remain unresolved, they contribute to more than 90% of the similarity score. Consequently, the differences in the architectures of the two proteins caused by the low complexity regions in the N- and C-terminal parts of the human protein reduces the similarity score only to a minor extent. Resolving the overlaps in the Pfam-/SMART layer removes this buffering effect. The low complexity regions present only in the human protein (Q6IQ22) increase in their weight, and thus the architecture differences result in a lower FAS score. Grey lines indicate the length of the respective proteins, where the scale is given below each architecture. Features that contribute to the representative architecture are indicated with boxes.

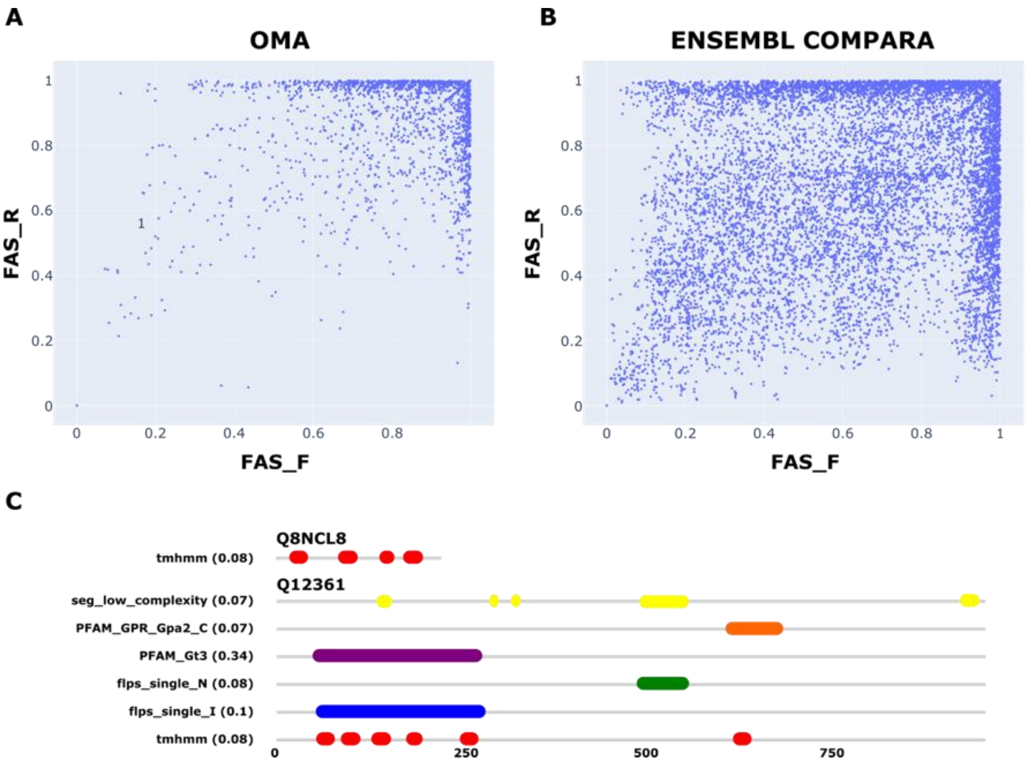

Figure S8: Analysis of the asymmetry between the forward (FAS_F) and the reverse FAS score (FAS_R). Each dot represents a human – yeast ortholog pair (OP_{h-y}) and we computed the FAS_F (human as reference) and the FAS_R (yeast as reference). We distinguish the OP_{h-y} with asymmetric scores ($\Delta FAS \geq 0.1$) into three categories: In C1, the architectures differ due to features confined to the human architecture, and an OP_{h-y} is assigned to C1 if FAS_R > 0.9. In C2, architectures differ due to features confined to the yeast architecture, and an OP_{h-y} is assigned to C2 if FAS_F > 0.9. In C3 both architectures comprise features not seen in the respective other architecture. We assign an OP_{h-y} to C3 if FAS_F < 0.9 and FAS_R < 0.9. (A) Orthology inferred with OMA (2,595 OP_{h-y}). C1: 764 OP_{h-y} (29%); C2: 469 OP_{h-y} (18%); C3: 259 OP_{h-y} (10%). (B) Orthology inferred with Ensembl Compara (12,676 OP_{h-y}). 2,715 OP_{h-y} (21%); C2: 2,316 OP_{h-y} (18%); C3: 3,856 OP_{h-y} (30%). (C) Example of an OP_{h-y} with asymmetric FAS_F and FAS_R scores. The proteins Q8NLC8 (human) and Q12361 (yeast) share only the presence of an N-terminal set of transmembrane domains.

O14521 (Human)

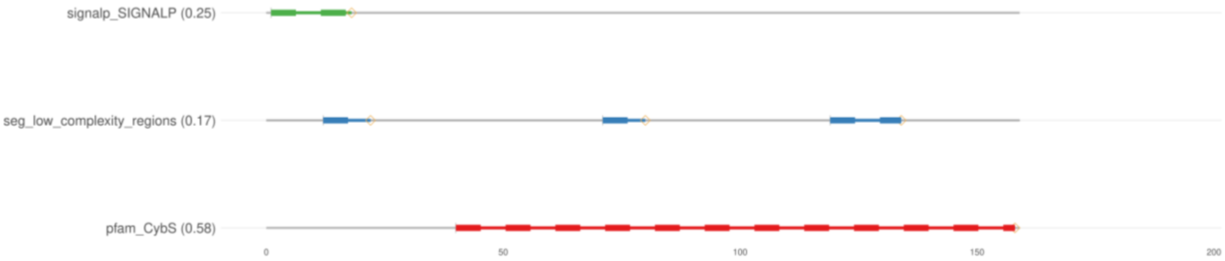

Q08749 (Yeast)

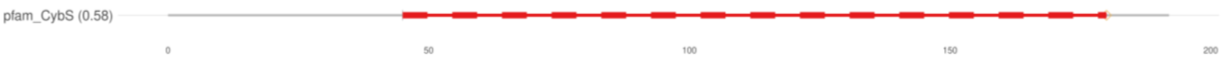

Figure S9: An example of nested feature architectures. The yeast protein (Uniprot-ID: Q08749) harbors only a Pfam CybS domain. The human protein (Uniprot-ID: O14521) carries additionally an N-terminal signal peptide as well as three low complexity regions. This difference results in a lower FAS score when using the human protein as the reference, while it is close to 1 when using the yeast protein as the reference. Grey lines indicate the length of the respective proteins, where the scale is given below each architecture. Grey lines indicate the length of the respective proteins, where the scale is given below each architecture. Features that contribute to the representative architecture are indicated with boxes.

Q7Z2Y5 (Human)

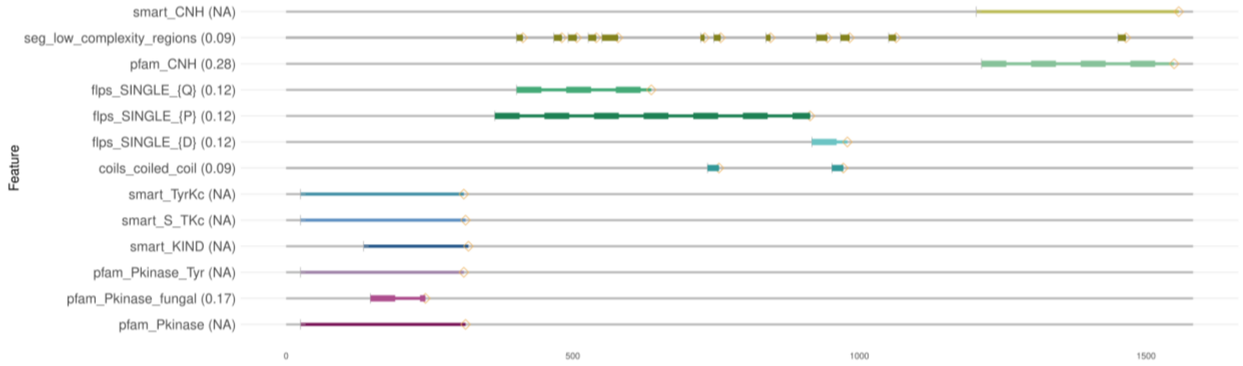

Q12469 (Yeast)

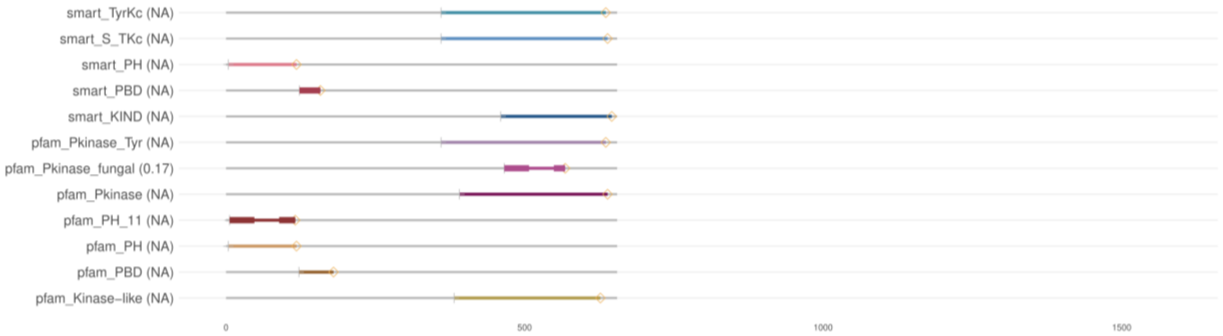

Figure S10: Feature architectures of Q7Z2Y5 and Q12469. Both proteins have a similar GO annotation (Schlicker Score: 0.97) but differ substantially in their feature architecture. On the feature architecture level, both proteins share only the presence of a tyrosine kinase domain, and correspondingly the FAS score is only 0.2. Features that contribute to the representative architecture are indicated with boxes.

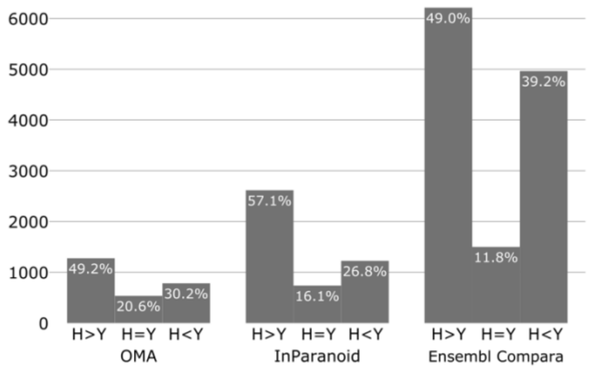

Figure S11: FAS score similarity distribution depends on the ortholog predictor. The histograms reveal the fraction of orthologs where the MLSA of the human protein comprises more features (H>Y), the same number of features (H=Y), or less features than the architecture of the corresponding yeast orthologs. For both OMA and InParanoid, only 30.2% and 26.8% of the yeast architectures comprise more features than that of their human orthologs. This number rises markedly to 39.2% for the Ensembl Compara ortholog pairs. At the same time the fraction with the same number of features is the lowest among the three tools.

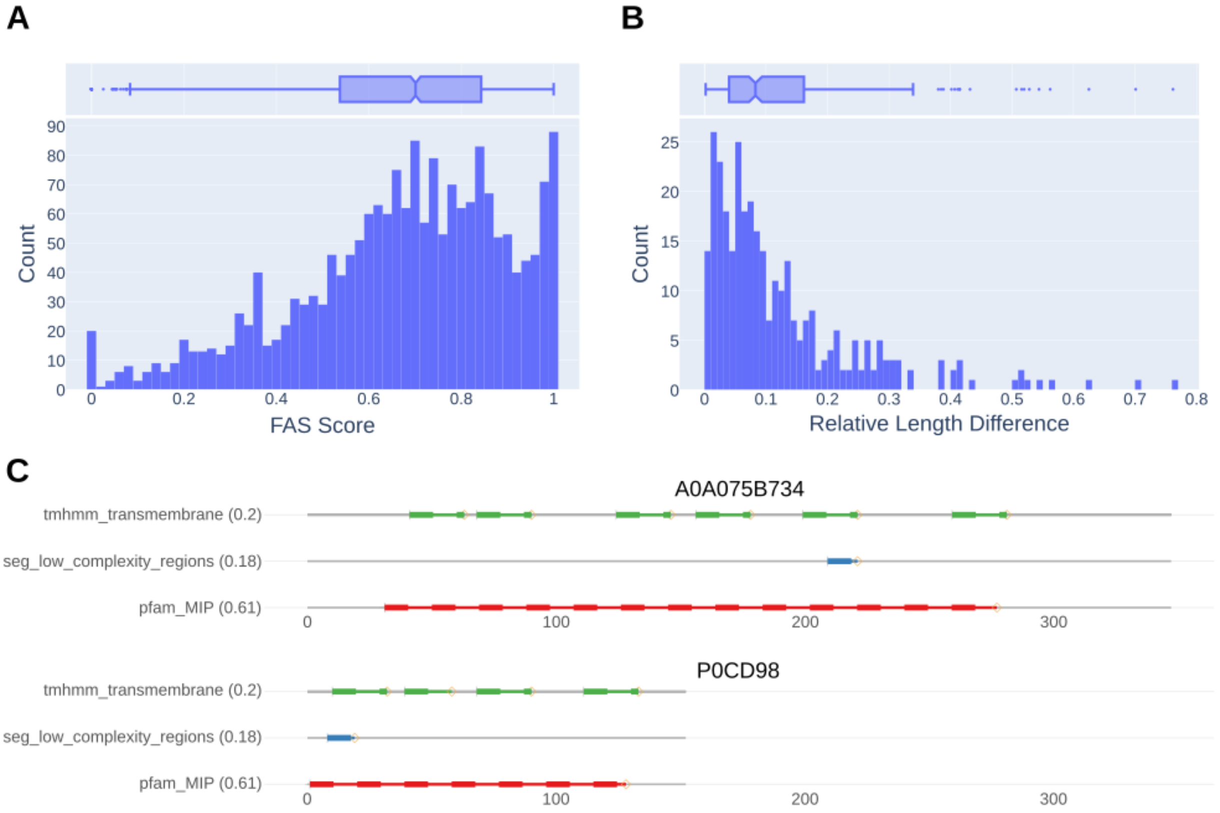

Figure S12: Characterization of 1,934 human proteins for which only Ensembl Compara assigned a yeast ortholog. (A) FAS score distribution of human – yeast ortholog pairs using the human protein as the reference. (B) Distribution of relative length differences (RLD) between human and yeast orthologs from (A) having a FAS score of at least 0.9. We computed the RLD as $|(l_{human} - l_{yeast})/max(l_{human}, l_{yeast})|$. (C) Feature architecture comparison of the human - yeast ortholog pair with the Uniprot IDs A0A075B734 and P0CD98. The two proteins are both annotated as aquaporins, which supports their assignment as orthologs by Ensembl Compara. The substantial length difference may explain why they have been missed by OMA and InParanoid, since the two algorithms require that 61% (OMA) or 50% (InParanoid) of the longer sequence is covered in the pair-wise alignment of two ortholog candidates (Remm, et al. 2001; Roth, et al. 2008). Please note, the analysis here is conservative because we compared only the lengths of the human and yeast proteins and not only the aligned part of the sequences. For all analyses, the mean bi-directional FAS score was used.

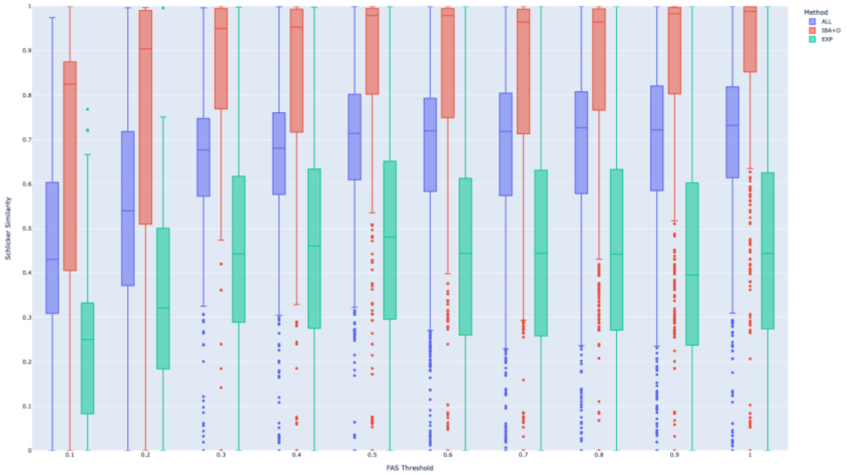

Figure S13: Semantic similarity of GO annotations decreases with decreasing feature architecture similarity. The box plots show the results when using all GO terms (blue; see also Fig. 5 in main text), only the subset with experimental confirmation (EXP; green), or the subset of GO terms where the annotation is based on phylogenetic evidence (IBA and IBD; red). While the general trend is independent of the evidence code used, the extent of Schlicker Similarity between the functional annotation of the orthologs is highest for IBA, and lowest for EXP.

Q9Y696 (Human)

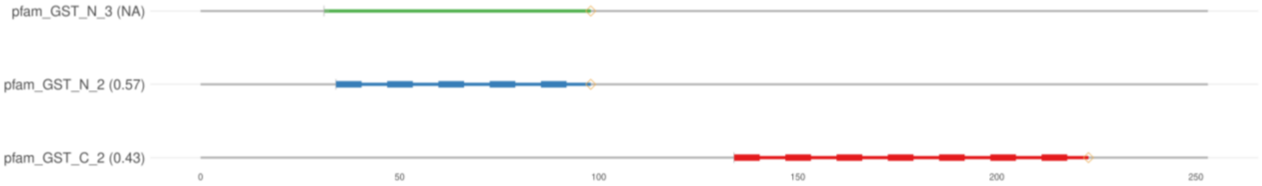

Q12390 (Yeast)

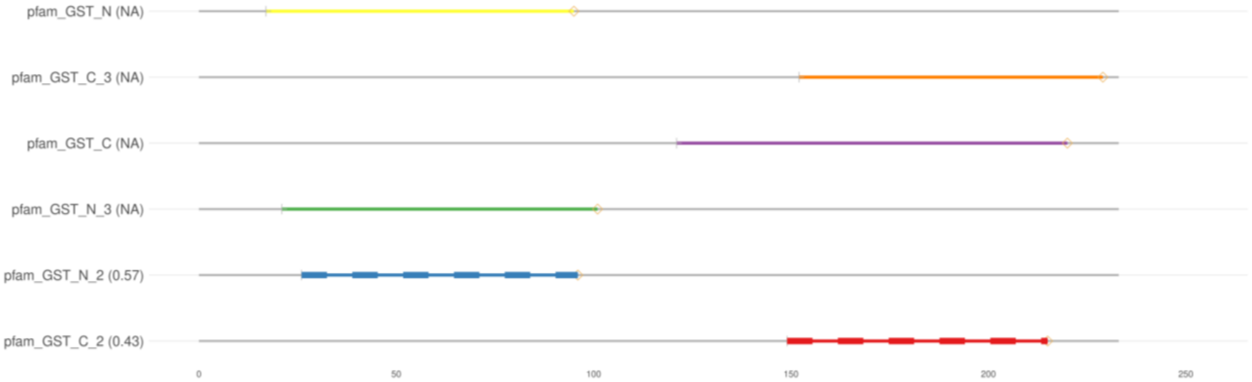

Figure S14: Feature architectures of Q9Y696 and Q12390. The two proteins differ in their GO annotation despite sharing the same feature architecture. Grey lines indicate the length of the respective proteins, where the scale is given below each architecture. Features that contribute to the representative architecture are indicated with boxes.

A

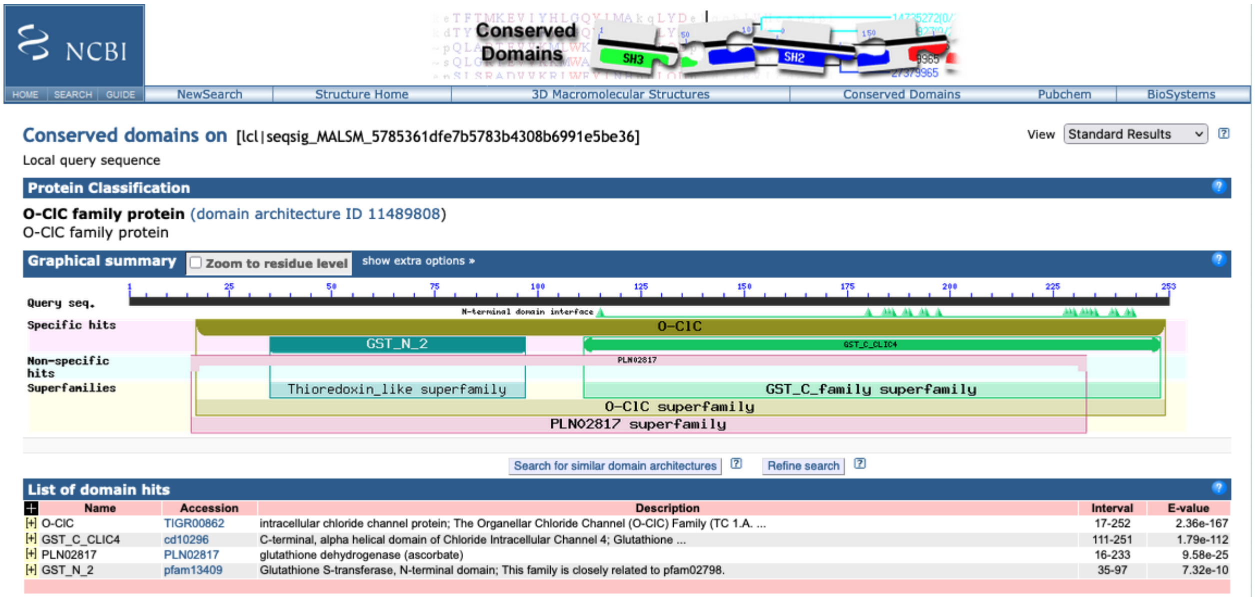

B

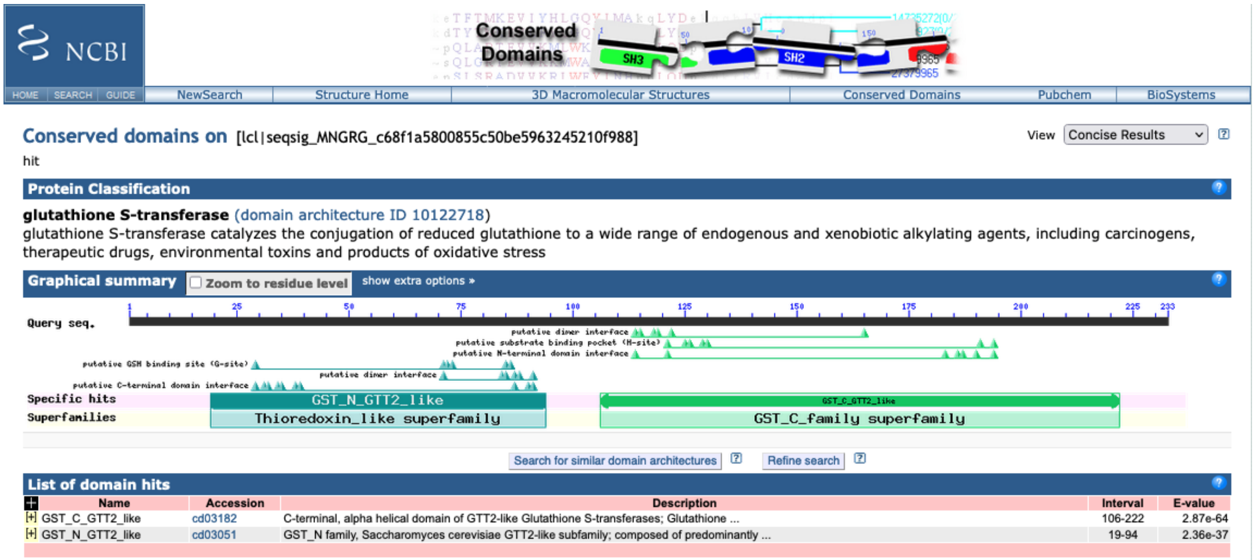

Figure S15: CD search result for Q9Y696 and Q12390. The human protein Q9Y696 is annotated as a chloride channel protein (A), the yeast protein Q12390 is annotated as a Glutathione S-transferase (B).

A

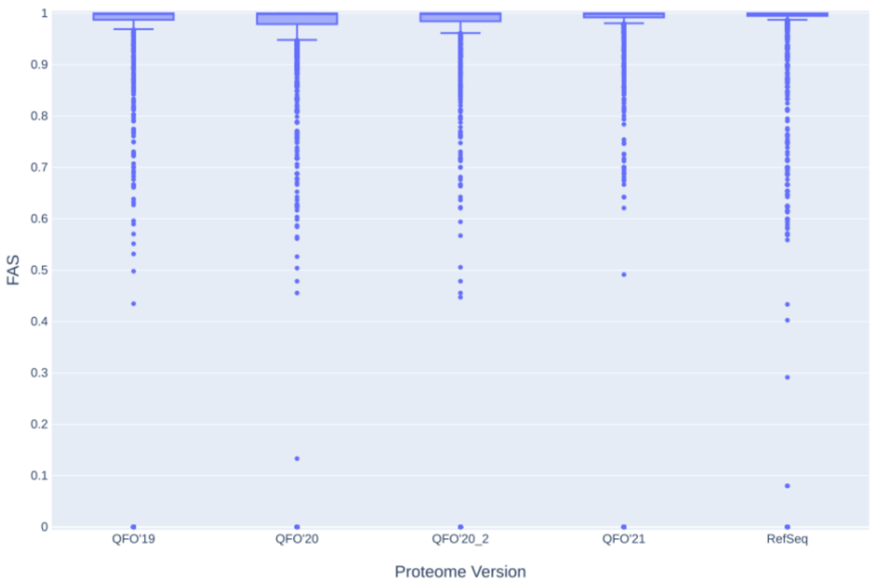

B

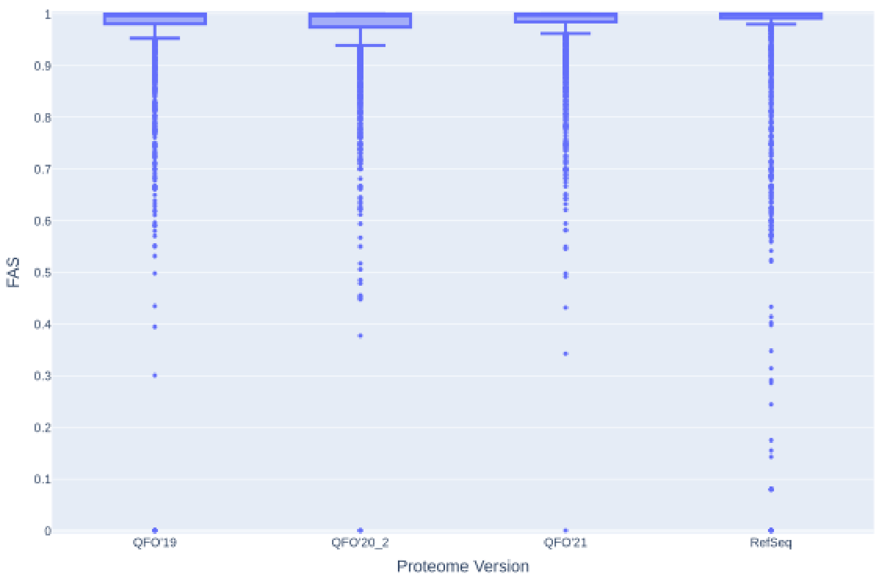

Figure S16: FAS score distribution between the *X. laevis* reference proteins and their orthologs in the *X. tropicalis* proteome versions. **(A)** The five box plots represent the FAS score distributions between between *X. laevis* and *X. tropicalis* orthologs separately for each *X. tropicalis* proteome. For better comparability, only the values for the 1,110 tetrapoda core genes are shown with orthologs present in all five *X. tropicalis* proteomes. **(B)** Same information as in (A) but without the QFO'20 proteome. This increased the number of analysed core genes to 2,056.

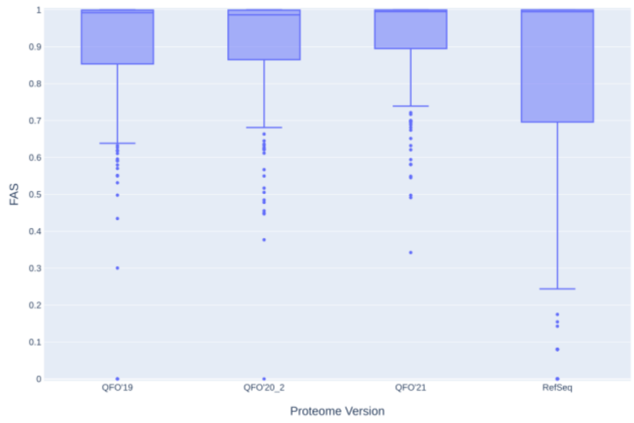

Figure S17: Feature architecture differences between different versions of the Xenopus tropicalis proteome without QFO'20. FAS score distributions for the 469 *X. laevis* – *Xt* ortholog pairs with a FAS score difference between the highest and the lowest scoring *Xt* ortholog version of at least 0.1.

Q5TAP6 (Human)

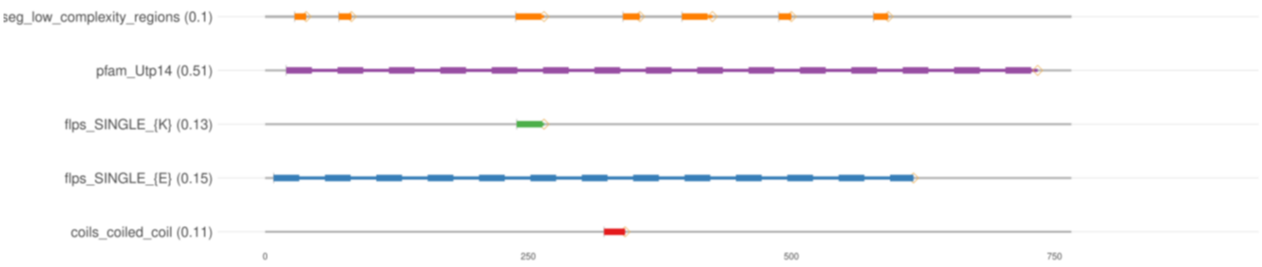

Q04500 (Yeast)

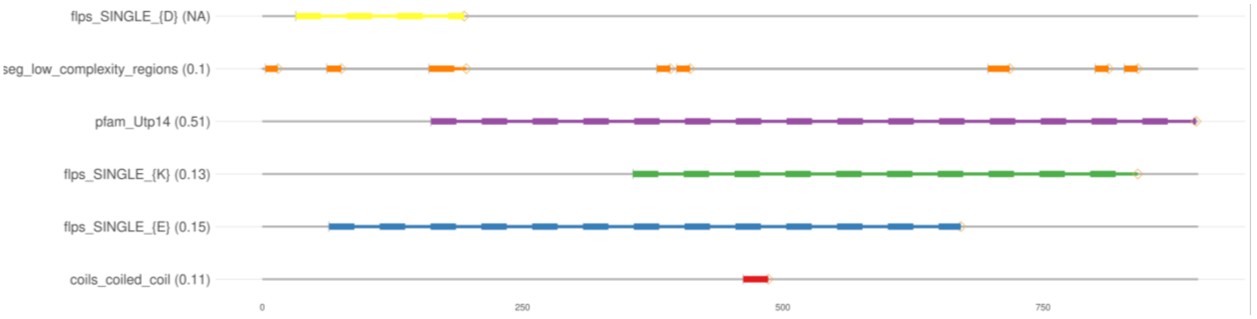

Figure S18: Feature architectures of Q5TAP6 and Q04500. The two proteins have a high similarity in their feature architecture, and both represent U3 small nucleolar RNA-associated protein 14. Still, the Schlicker score of the corresponding GO annotations is 0.0 (Table S2). Grey lines indicate the length of the respective proteins, where the scale is given below each architecture. Features that contribute to the representative architecture are indicated with boxes.

P38279

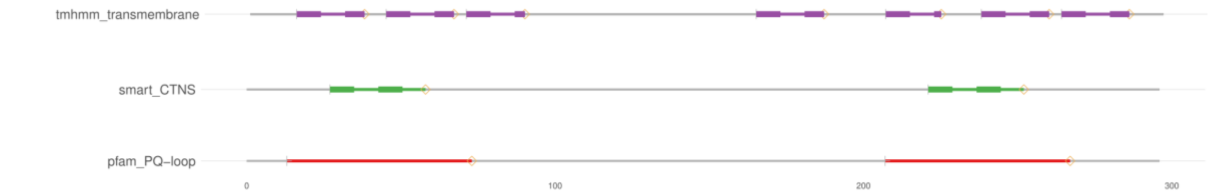

Q06328

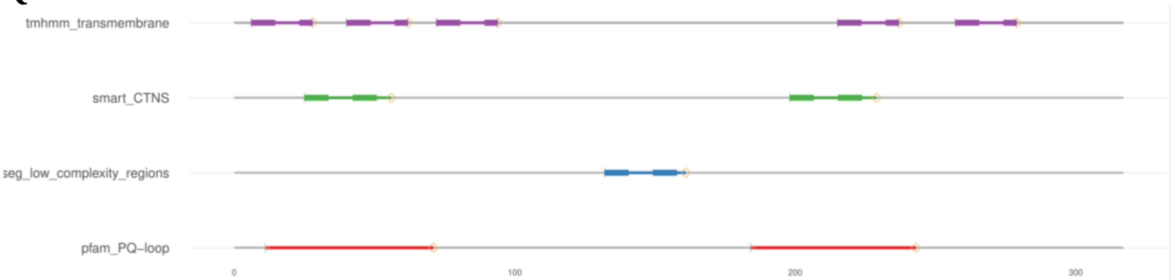

Q12010

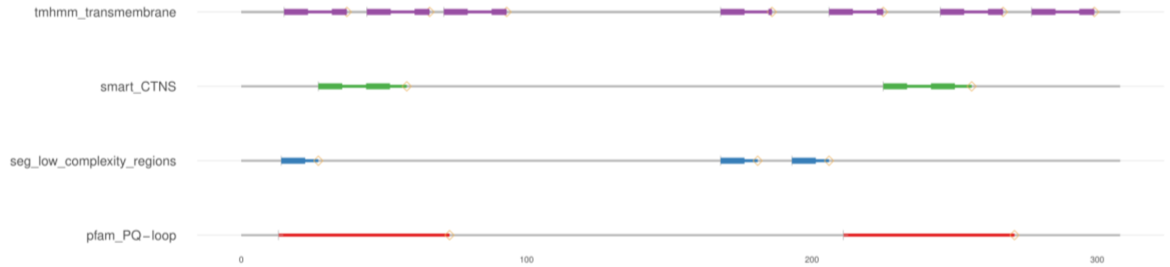

A1A4F0

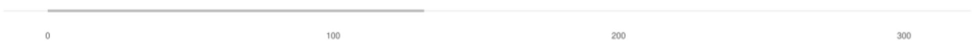

Figure S19: Examples for proteins that share the same GO annotation despite pronounced differences in their feature architectures. The three yeast proteins (P38279, Q06328, and Q12010) display almost identical feature architectures. Their human ortholog (A1A4F0) is about 200 amino acids shorter, and it is devoid of any features. Grey lines indicate the length of the respective proteins, where the scale is given below each architecture. Features that contribute to the representative architecture are indicated with boxes.

Supplementary tables

Table S1: Proteome sources

Proteome Version	#Proteins	Proteome Source
HUMAN QFO	20600	qfo_release-2020_04
YEAST QFO	6050	qfo_release-2020_04
XENTR QFO‘19	24386	qfo_release-2019_04
XENTR QFO‘20	9639	qfo_release-2020_04
XENTR QFO‘20_2	17272	qfo_release-2020_04_with_updated_UP000008143
XENTR QFO‘21	22514	qfo_release-2021_03
XENTR RefSeq#	45093	GCF_000004195.4_UCB_Xtro_10.0

The RefSeq proteome of X. tropicalis comprises also protein isoforms. In the course of the analysis, we selected the isoform that maximised the pair-wise FAS score to the X. laevis reference protein.

Table S2: Path complexities of the 10 human-yeast ortholog pairs for which the score maximization algorithm to resolve overlaps resulted in a lower score than the e-value-based approach

Human ID (Uniprot)	Yeast ID (Uniprot)	# of alternative paths (human)	# of alternative paths (yeast)	# of path evaluations (exhaustive)
Q12913	P25044	9216	6	55296
Q14554	Q12404	1703	6	10218
Q8IWX7	Q12118	216	291	62856
Q92824	P09232	630686	1	630686
Q92824	P25036	630686	1	630686
Q92824	P25381	630686	1	630686
Q96EQ0	P15705	391	5893568	2304385088
Q96EQ0	P38825	391	222870	87142170
Q9HD43	P25044	15008	6	90048
Q9Y4ES	P39933	4096	236520	968785920

Table S3: Curation of 80 human-yeast ortholog pairs with substantial discrepancies between FAS and Schlicker scores (|FAS – Schlicker| >= 0.75)

See file Dosch_TableS3.xlsx

Table S4: Molecular Function GO term annotation for the two ortholog groups (A1A4F0 & Q06328, P38279, Q12010) and (Q5TAP6 & Q04500)

Protein	GO Term ID	Description
A1A4F0	GO:0015174	basic amino acid transmembrane transporter activity
A1A4F0	GO:0015189	L-lysine transmembrane transporter activity
Q06328	GO:0015174	basic amino acid transmembrane transporter activity
P38279	GO:0015174	basic amino acid transmembrane transporter activity
Q12010	GO:0015174	basic amino acid transmembrane transporter activity
Q5TAP6	GO:0005515	protein binding
Q04500	GO:0003674	molecular function
Q04500	GO:0005524	ATP binding

Supplementary data

Supplementary data file 1 provides the orthology assignments for the 3460 tetrapod core genes in each of the five *X. tropicalis* proteome versions.

References

Remm M, Storm CE, Sonnhammer EL. (Remm:2001lr co-authors). 2001. Automatic clustering of orthologs and in-paralogs from pairwise species comparisons. J Mol Biol 314:1041-1052.

Roth A, Gonnet G, Dessimoz C. (Roth:2008lr co-authors). 2008. Algorithm of OMA for large-scale orthology inference. BMC Bioinformatics 9:518.
